# SQSTM1/p62 loss reverses the inhibitory effect of sunitinib on autophagy independent of AMPK signaling

**DOI:** 10.1038/s41598-019-47597-4

**Published:** 2019-07-31

**Authors:** Bolin Hou, Gang Wang, Quan Gao, Yanjie Wei, Caining Zhang, Yange Wang, Yuqing Huo, Huaiyi Yang, Xuejun Jiang, Zhijun Xi

**Affiliations:** 1 State Key Laboratory of Mycology, Institute of Microbiology, Chinese Academy of Sciences, Beijing, 100101 China; 2 University of Chinese Academy of Sciences, Beijing, 100039 China; 3 Department of Urology, Peking University First Hospital, Beijing, 100034 China; 4 Vascular Biology Center, Department of Cellular Biology and Anatomy, Medical College of Georgia, Augusta University, Augusta, 30912 Georgia USA; 5 CAS Key Laboratory of Pathogenic Microbiology and Immunology, Institute of Microbiology, Chinese Academy of Sciences, Beijing, 100101 China

**Keywords:** Macroautophagy, Cytoskeletal proteins

## Abstract

Sunitinib (ST), a multitargeted receptor tyrosine kinase inhibitor, has been demonstrated to be effective for the treatment of renal carcinoma. It has been reported that ST is involved in the mediation of autophagy; however, its regulatory role in the autophagic process remains controversial. Furthermore, the mechanism by which activated AMP-activated protein kinase (AMPK) negatively regulates autophagy remains nearly unexplored. In the present study, we revealed that ST inhibited AMPK activity and regulated autophagy in a cell type- and dose-dependent manner. In a number of cell lines, ST was demonstrated to inhibit H_2_O_2_-induced autophagy and the phosphorylation of acetyl-CoA carboxylase (ACC), whereas alone it could block the autophagic flux concurrent with increased expression of p62. An immunoprecipitation assay revealed that LC3 directly interacted with p62, whereas ST increased punctate LC3 staining, which was well colocalized with p62. Taken together, we reveal a previously unnoticed pathway for ST to regulate the autophagic process, and p62, although often utilized as a substrate in autophagy, plays a critical role in regulating the inhibition of ST in both basal and induced autophagy.

## Introduction

Macroautophagy (herein referred to as autophagy) is a process involving the bulk degradation of cytosolic components by autophagolysosomes^1^. It occurs in most eukaryotic cells at basal levels and is activated in response to the number of stimuli. It is well recognized that autophagy fulfills two major cellular functions: detoxification via waste removal and provision of protection in response to nutrient stress. Although often used as a substrate of autophagy, p62 was initially found as a signaling mediator residing in the late endosome and lysosome^2^. Accumulating evidence has indicated that p62 is a multifunctional adaptor protein that participates in the regulation of a series cellular functions, such as nutrient sensing, survival/apoptosis modulation, and signaling pathway activation^3,4^. Moreover, it is known to link the cellular degradation pathways, ubiquitin system, and autophagic machinery^5–7^. In vertebrates, p62 regulates the autophagic removal of protein aggregates and damaged organelles, including mitochondria, through its interaction with ubiquitin and the LC3 component of autophagy^8,9^. Although it is recognized to play an essential role in mediating selective autophagy, p62 is required for the unselected autophagic process, and its loss inhibits resveratrol (RSV)–induced autophagy^10^.

ST is a multitargeted receptor tyrosine kinase inhibitor, and it inhibits the activity of PDGFRs, c-KIT, FLT-3, and VEGFRs, all of which have been demonstrated to be important for cell proliferation, migration, and angiogenesis^11^. ST can induce both cell viability loss and cell senescence, and it can cause G1-S cell cycle arrest and the DNA damage response in OS-RC-2 cells^12,13^. Recently, ST has been demonstrated to mediate autophagy depending on the cell type. In both cardiac and PC12 cells, ST increases autophagic flux, whereas it induces incomplete autophagy in either renal or bladder cancer cells. In RCC 786-O cells, ST increases the level of phosphorylated EGFR, which may cause resistance to ST treatment in RCC^14^. In endometrial carcinoma, ST decreases either the basal or EGF-activated NFkB transcription^15^. Additionally, ST has been demonstrated to inhibit AMPK and cause myocyte cytotoxicity^16,17^.

AMPK, a key energy-surveillance kinase complex, contains three subunits: a catalytic, one scaffolding and a regulatory. Energy stress increased the levels of either ADP or AMP (compared with ATP), and their augment functioned as an index of energy deprivation. AMPK was firstly found to be a kinase that directly inhibited ACC through increasing its phosphorylation; it also functions in multiple ways to influence cellular metabolism, and its activation is upregulated responsive to various stress conditions with an increased ratio of AMP to ATP. Through enhancing Thr172 phosphorylation of the catalytic subunit and inhibiting dephosphorylation of Thr172, AMP binding was found to increase the activity of AMPK. Mammalian target of rapamycin complex 1 (mTORC1) is a multiprotein complex consisting of mTOR, raptor, mLST8, and PRAS40, and AMPK demonstrated to inhibit mTORC1 via activating tuberous sclerosis complex 2 (TSC2) and directly reducing the phosphorylation of raptor^18,19^. Therefore, AMPK has been thought to trigger autophagy through an indirect mechanism by inhibiting mTORC1 activity or directly binding to ULK1, which is a serine/threonine kinase and also known as ATG1^20,21^. However, Shang *et al*. observed that nutrient starvation simulates the autophagic response mediated by ULK1 dephosphorylation and its dissociation from AMPK^22^. They further suggested that AMPK might have dual roles in the regulation of autophagy depending on the nutrient condition. Indeed, compound C, a pharmacological AMPK inhibitor that efficiently blocks the metabolic actions of AMPK, has been demonstrated to induce the autophagic process in different cancer cell lines^23^.

Here, we show that ST treatment alone can either inhibit or induce autophagy depending on the cell type and its concentration. ST was demonstrated to inhibit AMPK activity, upregulated p62 expression and abolished the H_2_O_2_-induced autophagic flux. While deprivation of p62 reversed the inhibitory effect of ST on basal autophagy, it no longer blocked the H_2_O_2_-activated autophagic flux in p62-depleted cells.

## Results

### Inhibition of autophagy increases the cleavage of PARP-1 induced by ST

As an approved drug for RCC treatment^24^, ST expectedly reduced the cell viability of both 786-O and ACHN in a dose-dependent manner (Fig. S1a). The cytotoxic effect of ST was further confirmed by the observation that the cleavage of PARP-1, which serves as a marker of cells undergoing apoptosis^25^, was induced by ST (Fig. S1b). The addition of 3-MA, a widely used inhibitor of early autophagy^26^, increased PARP-1 cleavage, while the usage of CQ, which inhibited the fusion between autophagosomes and lysosomes^27^, further augmented ST/3-MA-induced caspase-dependent apoptosis (Fig. S1b). Moreover, deprivation of either LC3 or Beclin 1 increased the cleavage of PARP-1 (Fig. S1c–e), suggesting that autophagy is involved in the ST-induced apoptotic process. Two different LC3 or Beclin 1 small interfering RNAs (siRNAs) were used to independently knock down the expression of either LC3 or Beclin 1 (Fig. S3a,b). In addition, p62, a substrate of autophagic degradation with increased expression during autophagy inhibition^28^, appeared to be upregulated in both LC3- and Beclin 1-depleted cells (Fig. S1c–e).

### A high dose of ST inhibits the autophagic flux and increases p62 expression

**Figure 1:**
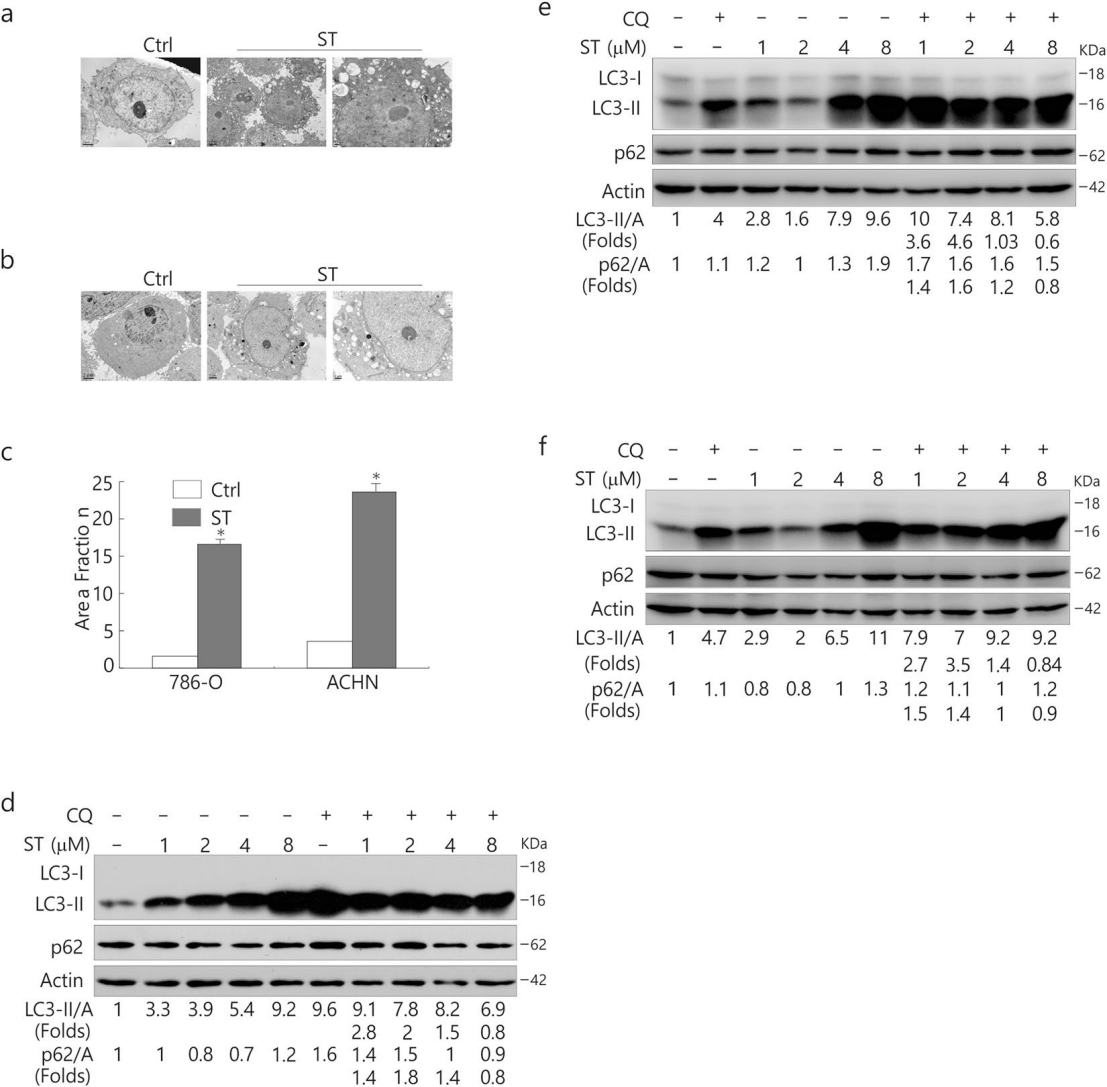
A high dose of ST inhibits the autophagic flux and increases p62 expression. (**a**–**c**) Electron microscopy was performed in 786-O (**a**) and ACHN (**b**) cells following ST treatment (4 μM/L) for 4 h. The morphometric analysis of the area fraction between autophagosomes and cytoplasm was calculated using Photoshop software (**c**). The area ratio data were non-normally distributed and are presented as the mean of at least 10 cells counted for each group. ACHN (**d**), 786-O (**e**) and HeLa (**f**) cells were treated with ST (0–8 μM/L) for 4 h in the presence or absence of CQ (ACHN: 10 μM/L; HeLa: 15 μM/L; 786-O: 20 μM/L). The cells were lysed and subjected to immunoblotting (8–13% PAGE) with the indicated antibodies. Densitometry was performed for quantification, and the adjusted ratio of LC3-II and p62 to actin is presented under the blots. The results were similar among experiments repeated at least twice. (Control: Ctrl; Beclin1: Bec1; Actin: A).

Recent studies have revealed contradictory results about the involvement of ST in autophagy^29^; thus, we subsequently examined the autophagic flux upon ST challenge in both 786-O and ACHN cells. Through electron microscopy, we observed an obvious accrual of membrane vacuoles in either ST-treated ACHN or 786-O cells, and cytosolic components were sequestered in some of those vacuoles in comparison to the control (Fig. 1a–c). The immunoblotting analysis revealed that ST treatment increased the ratio of LC3-II to actin relative to control cells in a concentration-dependent manner (Fig. 1d,e). Unexpectedly, we found that a high dose of ST (8 μM/L) appeared to completely inhibit the autophagic flux since CQ failed to further accumulate LC3-II in both cell lines (Fig. 1d,e), whereas treatment with 4 μM/L ST abolished the autophagic flux only in 786-O cells (Fig. 1e). In contrast to ACHN cells, in which it either increased or decreased the level of p62 (Fig. 1d), ST solely augmented the expression of p62 in 786-O cells (Fig. 1e). Additionally, a high dose of ST (8 μM/L) also blocked autophagy in HeLa cells (Fig. 1f). Similar to ACHN cells, ST could increase or decrease the level of p62 in HeLa cells. However, ST at a dose of 8 μM/L was found to increase the expression of p62 in all three cell lines (Fig. 1d–f). The aforementioned results indicated that ST regulated autophagy in a dose- and cell type-dependent manner, and it could inhibit the autophagic flux under certain conditions.

### ST inhibits H_2_O_2_-induced autophagy concurrent with the downregulation of AMPK activity

**Figure 2:**
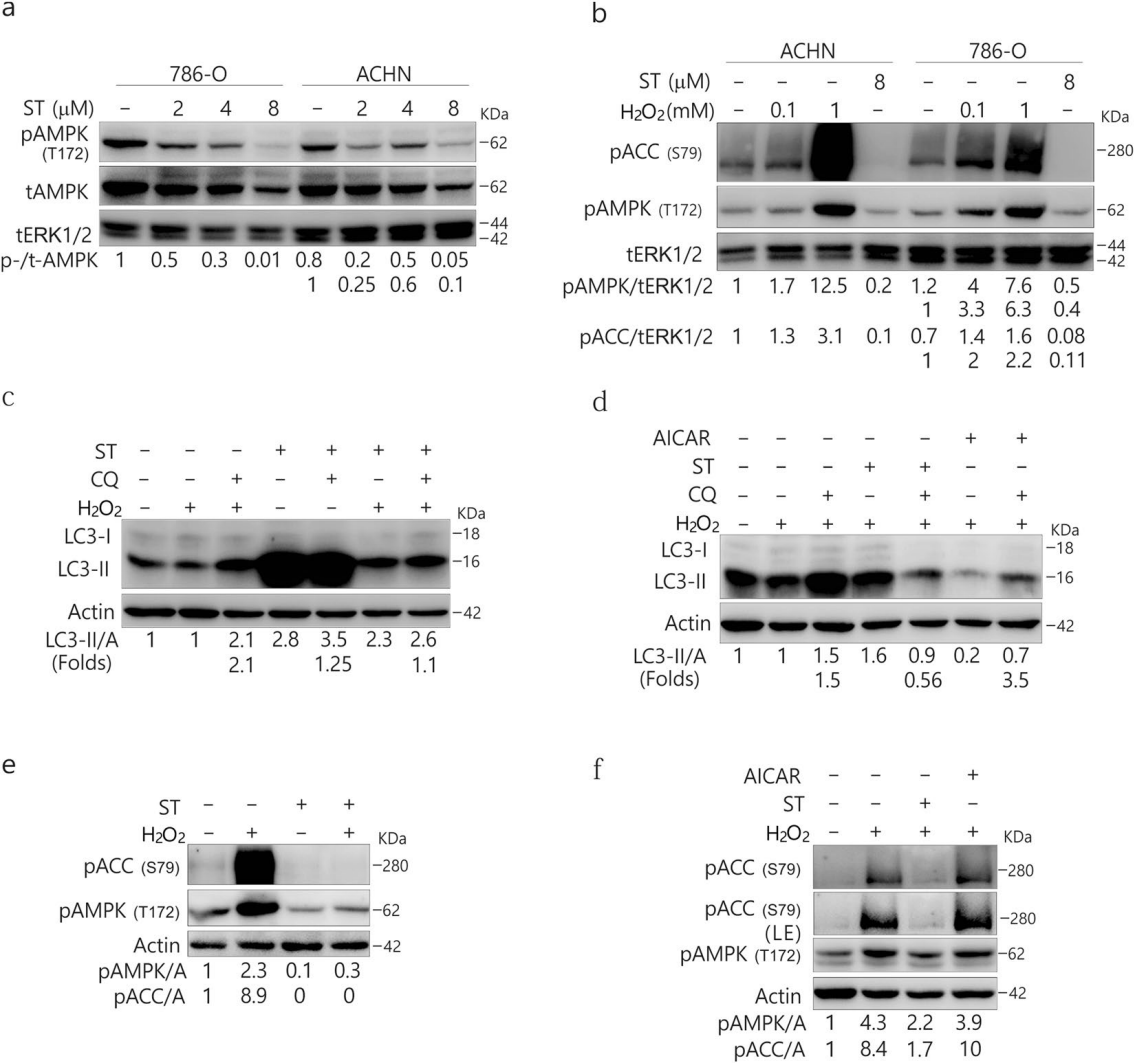
ST inhibits the phosphorylation of AMPK and ACC and blocks H_2_O_2_-induced autophagy concurrent with the downregulation of AMPK activity. 786-O and ACHN (**a**,**b**) cells were treated with ST (0–8 μM/L) or H_2_O_2_ (0.1, 1 mM/L; 2 h) for up to 4 h, and the cells were lysed and subjected to immunoblotting (8–13% PAGE) with the indicated antibodies. tERK1/2 was used as a loading control. The results were similar among experiments repeated at least three times. ACHN (**c**,**e**) and 786-O (**d**,**f**) cells were treated with H_2_O_2_ (ACHN: 0.5 mM/L; 786-O: 0.1 mM/L) with or without ST (8 μM/L), or AICAR (0.5 mM/L) in the presence or absence of CQ (ACHN: 10 μM/L; 786-O: 20 μM/L) for 2 h. Cells were lysed and subjected to immunoblotting (8–13% PAGE) with the indicated antibodies. Actin was used as a loading control. The results were similar among experiments repeated at least twice. (Actin: A).

Former studies have been revealed that ST inhibits the phosphorylation of AMPK and causes myocyte cytotoxicity^16,17^. Consistently, we observed that ST markedly decreased AMPK phosphorylation in all three tested cell lines (Figs 2a, S2a). In contrast, H_2_O_2_ markedly increased the phosphorylation of ACC, the AMPK substrate and indicator of AMPK activity^30^ (Figs 2b, S2b), and ST abolished the H_2_O_2_-induced phosphorylation of ACC (Figs 2b,e, S2b,e). To investigate the inhibitory mechanism of ST on autophagy, we determined the autophagic flux following treatment of the cells with H_2_O_2_ in the presence or absence of ST. Although either H_2_O_2_ or ST induced autophagy, their combination failed to further stimulate the autophagic process in ACHN cells (Fig. 2c). In 786-O cells, ST abolished the H_2_O_2_-activated autophagic flux as CQ was unable to accumulate LC3-II in H_2_O_2_/ST-treated cells (Fig. 2d). In HeLa cells, ST also completely inhibited H_2_O_2_-induced autophagy (Fig. S2c). The 5-aminoimidazole-4-carboxamide1-β-D-ribofuranoside (AICAR), an agonist of AMP-activated protein kinase (AMPK), enhanced H_2_O_2_-induced autophagy in 786-O cells (Fig. 2d). Notably, ST abolished H_2_O_2_-activated phosphorylation of ACC (Figs 2e,f, S2d), whereas AICAR increased the activity of AMPK (Fig. 2f). Therefore, ST was able to mediate the H_2_O_2_-activated autophagic process through AMPK/ACC signaling. Given that ST alone can activate autophagy, ST is likely to play a dual role in the regulation of autophagy in a dose-, cell type- and context-dependent manner.

### ST increases the expression of p62 and enhances its colocalization with LC3

**Figure 3:**
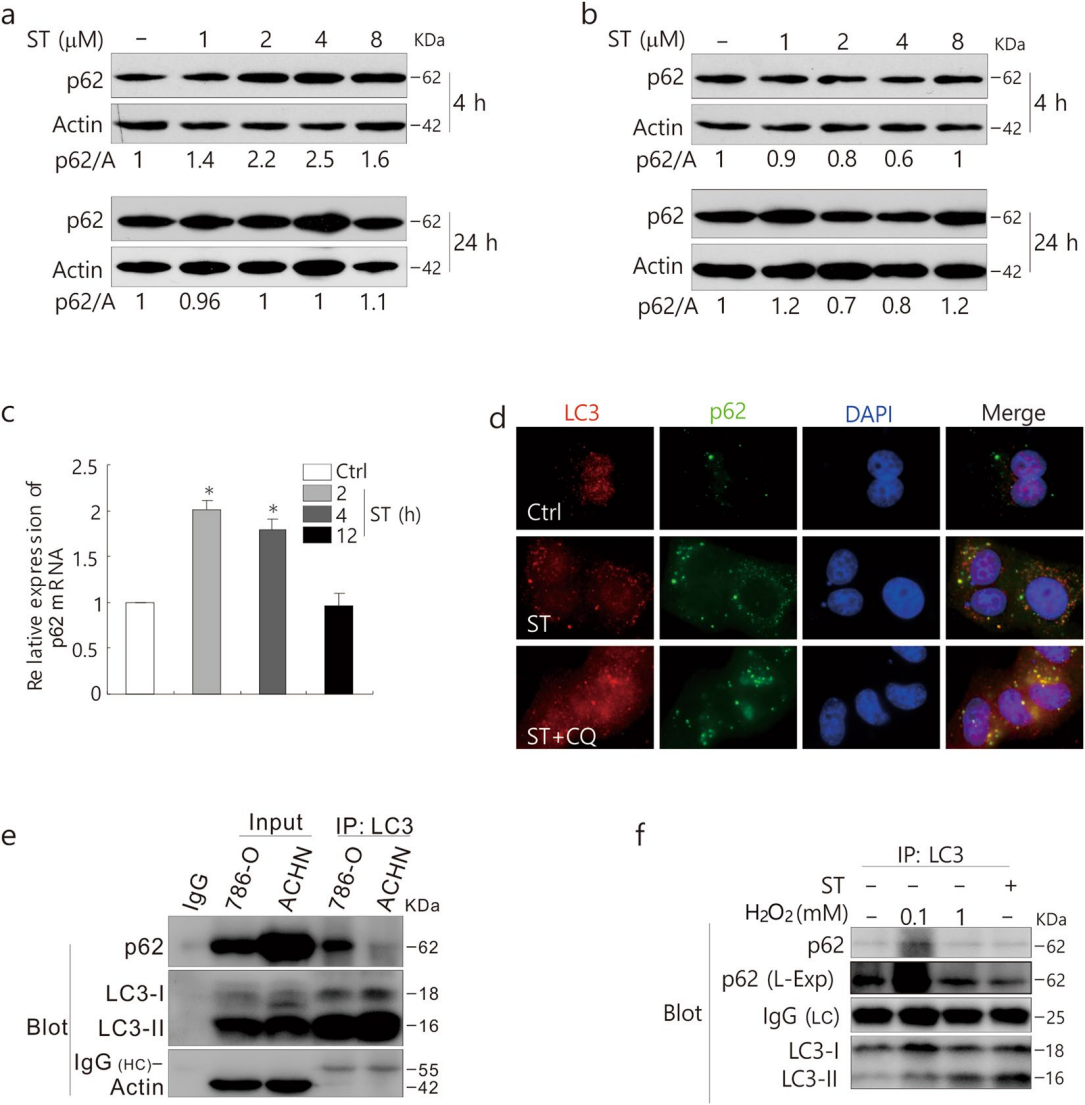
ST increases the expression of p62 in 786-O cells. 786-O (**a**) and ACHN (**b**) cells were treated with ST (0–8 μM/L) for up to 24 h. Cells were lysed and subjected to immunoblotting (8–13% PAGE) with the indicated antibodies. Actin was used as a loading control. **(c)** qPCR was performed to detect the mRNA expression of p62 following ST treatment for up to 24 h in 786-O cells. **(d)** 786-O cells were split onto coverslips, cultured overnight, and treated with ST (4 μM/L) or together with CQ (15 μM/L). They were then fixed with 4% paraformaldehyde, and images were obtained by fluorescence microscopy after labeling with anti-LC3 and p62 antibodies and staining with DAPI. **(e)** IgG2b (rabbit) was used as a negative control. ACHN and 786-O cells were lysed and precipitated using the antibody against LC3. **(f)** Following treatment with ST (8 μM/L: 4 h) or H_2_O_2_ (0.1, 1 mM/L: 2 h) for up to 4 h, 786-O cells were lysed and precipitated using the antibody against LC3. The immunoprecipitates were resolved by electrophoresis and probed by immunoblotting (8–13% PAGE) with the indicated antibodies. The results were similar among experiments repeated at least twice. (L-Exp: long exposure; HC: heavy chain; LC: light chain; Actin: A; Input: whole cell lysate).

Generally, p62 is considered to be a substrate in autophagic degradation, and the activation of autophagy usually causes a decrease in the p62 level. In comparison to ACHN cells, ST consistently increased the expression of p62 in 786-O cells (Fig. 3a,b). Real-time quantitative PCR (qPCR) results demonstrated that ST increased the transcriptional expression of p62 at both the 2 and 4 h time points (Fig. 3c and Table 1). The immunostaining assay revealed that ST increased punctate LC3 staining, which colocalized well with p62, and CQ addition further increased punctate LC3 staining in ST-treated cells and enlarged the dot staining of p62 (Fig. 3d). Consistent with a former report^31^, we observed a direct interaction between LC3 and p62 (Fig. 3e), whereas IgG, which was used as a negative control, failed to pull-down p62 (Fig. 3e). Moreover, the immunoprecipitation results displayed that either ST or H_2_O_2_ regulated the interaction between LC3 and p62 (Fig. 3f).

**Table 1:** Primer sequences. p62 and β-actin were defined as forward primer and reverse primer, respectively.

Gene	Primer	Nucleotide
p62	Forward (5′ → 3′)	CATCGGAGGATCCGAGTGTG
	Reverse (5′ → 3′)	TTCTTTTCCCTCCGTGCTCC
β-actin	Forward (5′ → 3′)	GCCTGACGGCCAGGTCATCAC
	Reverse (5′ → 3′)	CGGATGTCCACGTCACACTTC

### Deprivation of p62 reverses the inhibitory effect of ST on autophagy

**Figure 4:**
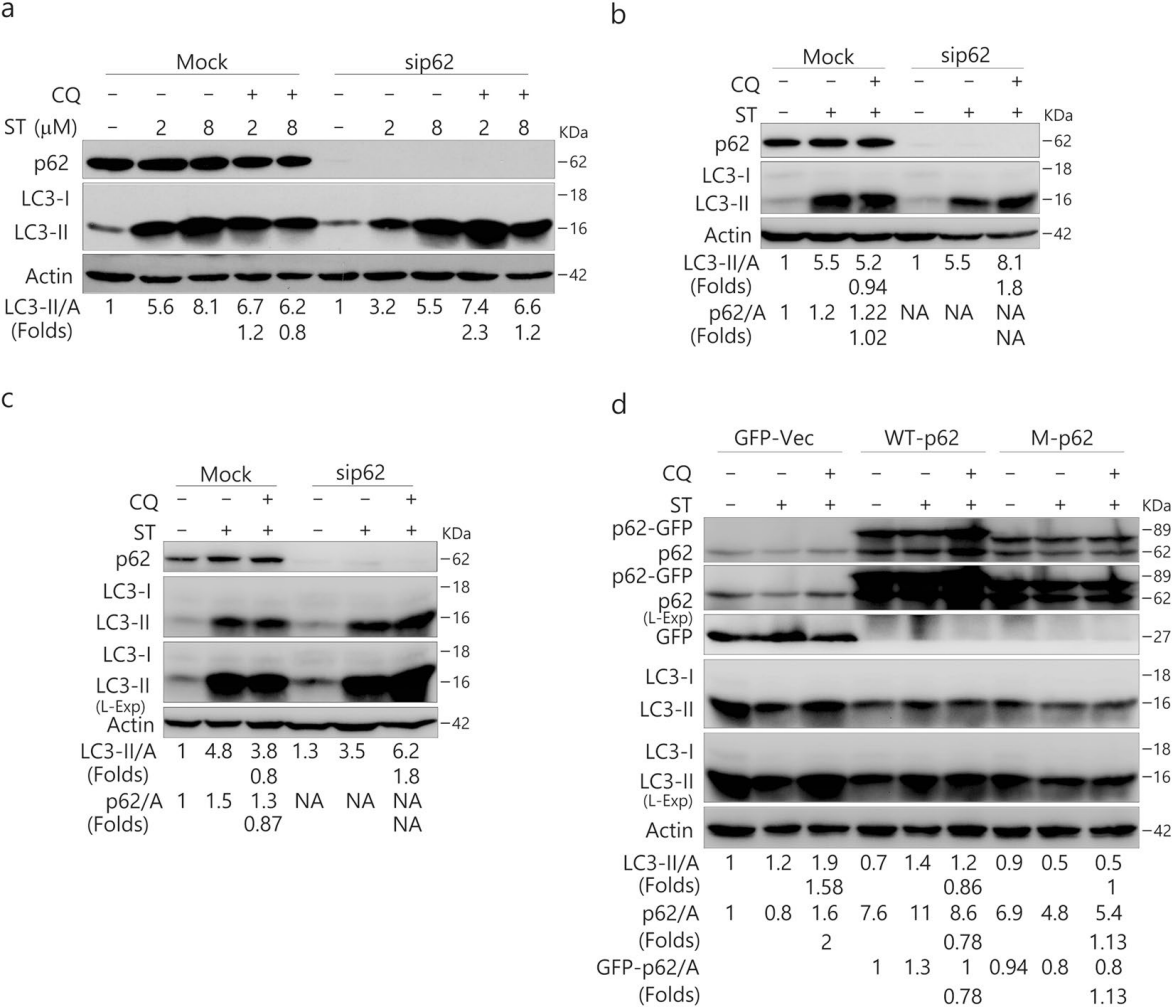
Deprivation of p62 reverses the inhibition of ST-induced autophagy. 786-O (**a**), ACHN (**b**) and HeLa (**c**) cells were transfected with the p62 siRNAs (sc-29679) for 48 h. HEK293T cells with either wild type (WT) or mutated p62 plasmid (**d**). The lysates were analyzed by immunoblotting (8–13% PAGE) following ST (8 μM/L or as indicated) treatment for 4 h in the presence or absence of CQ (ACHN: 10 μM/L; HeLa: 15 μM/L; 786-O: 20 μM/L; HEK293T 20 μM/L). Actin was used as the loading control. The results were similar among experiments repeated three times. (L-Exp: long exposure; Actin: A).

Since the knockdown of p62 was shown to inhibit resveratrol (RSV)–induced autophagy^10^ and ST increased the levels of p62, we speculated that p62 might be required for ST-activated autophagy. Unexpectedly, knockdown of p62 did not inhibit the ST (2 μM/L)-induced autophagic flux in 786-O cells (Fig. 4a). In contrast, its loss reversed the inhibitory effect of ST (8 μM/L) on autophagy (Fig. 4a). Similar results were also obtained in p62-depleted ACHN and HeLa cells (Fig. 4b,c).

To confirm the inhibitory effect of p62 on ST-induced autophagy, we transfected HEK293T cells with either wild type (WT) or mutated p62 plasmid (Fig. 4d). As expected, overexpression of WT p62 completely inhibited the ST-induced autophagic flux, as CQ failed to accumulate LC3-II in these cells (Fig. 4d), whereas the mutated p62, which lacks the ubiquitin (UB) binding domain, displayed less inhibition of ST-activated autophagy compared with WT p62 (Fig. 4d).

### ST fails to abolish the H_2_O_2_-autophagic flux in p62-depleted cells

**Figure 5:**
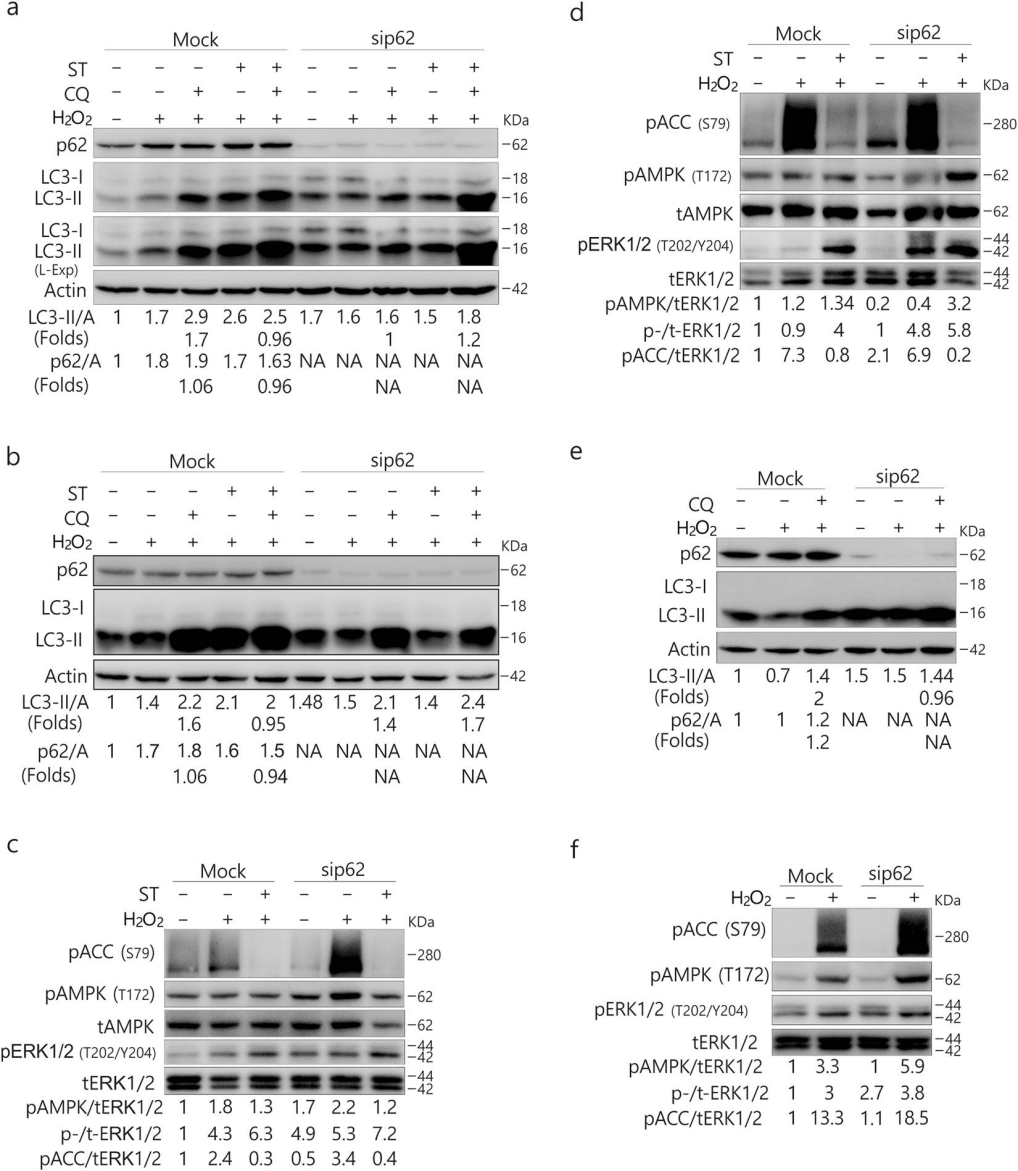
Depletion of p62 inhibits H_2_O_2_-induced autophagy and reverses the inhibitory effect of ST on H_2_O_2_-dependent autophagic flux. 786-O (**a**,**c**) and HeLa (**b**,**d**–**f**) cells were transfected with p62 siRNAs (sc-29679) for 48 h. The lysates were analyzed by immunoblotting (8–13% PAGE) following treatment with H_2_O_2_ (A, B, C and D: 0.1 mM/L; C and F: 0.5 mM/L) with or without ST (8 μM/L) for 2 h in the presence or absence of CQ (786-O: 20 μM/L; HeLa: 15 μM/L). The results were similar among experiments repeated twice. (L-Exp: long exposure; Actin: A).

As the depletion of p62 could reverse the inhibitory effect of ST on autophagy, we next determined whether p62 also played a regulatory role in the inhibitory effect of ST on H_2_O_2_-induced autophagy. Compared with the Mock-control, H_2_O_2_-induced autophagic flux was greatly inhibited in p62-depleted 786-O cells (Fig. 5a**)**, suggesting the likely requirement for p62 in H_2_O_2_-induced autophagy under these circumstances. In contrast to the Mock-control, ST failed to completely inhibit the H_2_O_2_-activated autophagic flux in p62-depleted 786-O cells (Fig. 5a**)**. Similar results were also obtained in HeLa cells (Fig. 5b). Notably, H_2_O_2_ combined with ST induced normal autophagy in p62-deprived HeLa cells compared with the Mock-control ones (Fig. 5b). Using a different siRNA, we obtained similar results in 786-O cells (Fig. S3c,d). Moreover, we observed that ST was able to abolish H_2_O_2_-increased phosphorylation of ACC in either Mock-control or p62-depleted cells (Fig. 5c,d). In p62-depleted HeLa cells, H_2_O_2_ at a dose of 0.5 mM/L failed to induce autophagy concurrent with an increase in the phosphorylation of ACC (Fig. 5e,f), suggesting that p62 could mediate H_2_O_2_-induced autophagy in a dose-dependent manner (Fig. 5b,e). Together with the findings shown in Fig. 5c,d, these results indicated that p62 could function downstream of AMPK signaling to regulate the autophagic process. While p62 silencing alone increased H_2_O_2_-induced phosphorylation of both extracellular signal-regulated kinase (ERK)1/2 and AMPK, its loss mediated basal ACC phosphorylation in a cell type-dependent manner (Fig. 5d,e). In 786-O cells, the deprivation of p62 decreased basal phosphorylation of ACC (Fig. 5d), whereas p62 loss increased the phosphorylated ACC in HeLa cells (Fig. 5e). Uncoupling between the phosphorylation of AMPK and ACC was clearly present. For example, while ST blocked the H_2_O_2_-induced phosphorylation of ACC in both cell lines (Fig. 5d,e), its presence increased H_2_O_2_-induced AMPK phosphorylation in HeLa cells (Fig. 5e). Given that p62 loss increased the phosphorylation of ERK1/2 (Fig. 5d,e), we speculated that mitogen-activated protein kinases (MAPK) signaling might play a role in either ST- or H_2_O_2_-induced autophagy.

**Figure 6:**
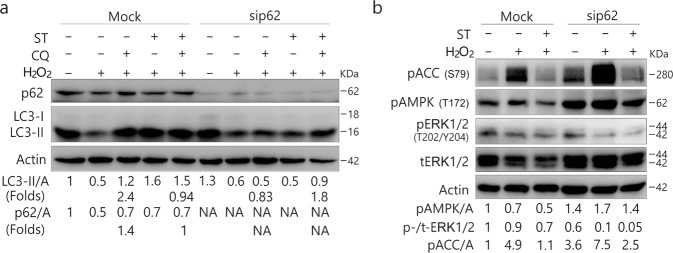
Detailed evaluation of the regulatory role of p62 in H_2_O_2_/ST-mediated autophagy. 786-O (**a**,**b**) cells were transfected with p62 siRNAs (sc-29679) for 48 h. The lysates were analyzed by immunoblotting (8–13% PAGE) following treatment with H_2_O_2_ (0.1 mM/L) with or without ST (8 μM/L) up to 4 h in the presence or absence of CQ (20 μM/L). The results were similar among experiments repeated twice.

To further explore the regulatory role of p62 in H_2_O_2_/ST-mediated autophagy, we prolonged the treatment of 786-O cells with H_2_O_2_ up to 4 h. As shown in Fig. 6a, ST failed to inhibit H_2_O_2_-activated autophagy in the p62-depleted cells, whereas it still abolished the H_2_O_2_-induced autophagic flux in Mock-control ones (Fig. 6a). In contrast to the 2 h time point, loss of p62 alone increased the phosphorylation of ACC and decreased the phosphorylation of ERK1/2 at the 4 h time point (Fig. 6b), suggesting that p62 regulated both activities of AMPK and ERK1/2 in a time-dependent manner.

### Inhibition of MEK/ERK signaling abolishes the ST-induced autophagic flux

**Figure 7:**
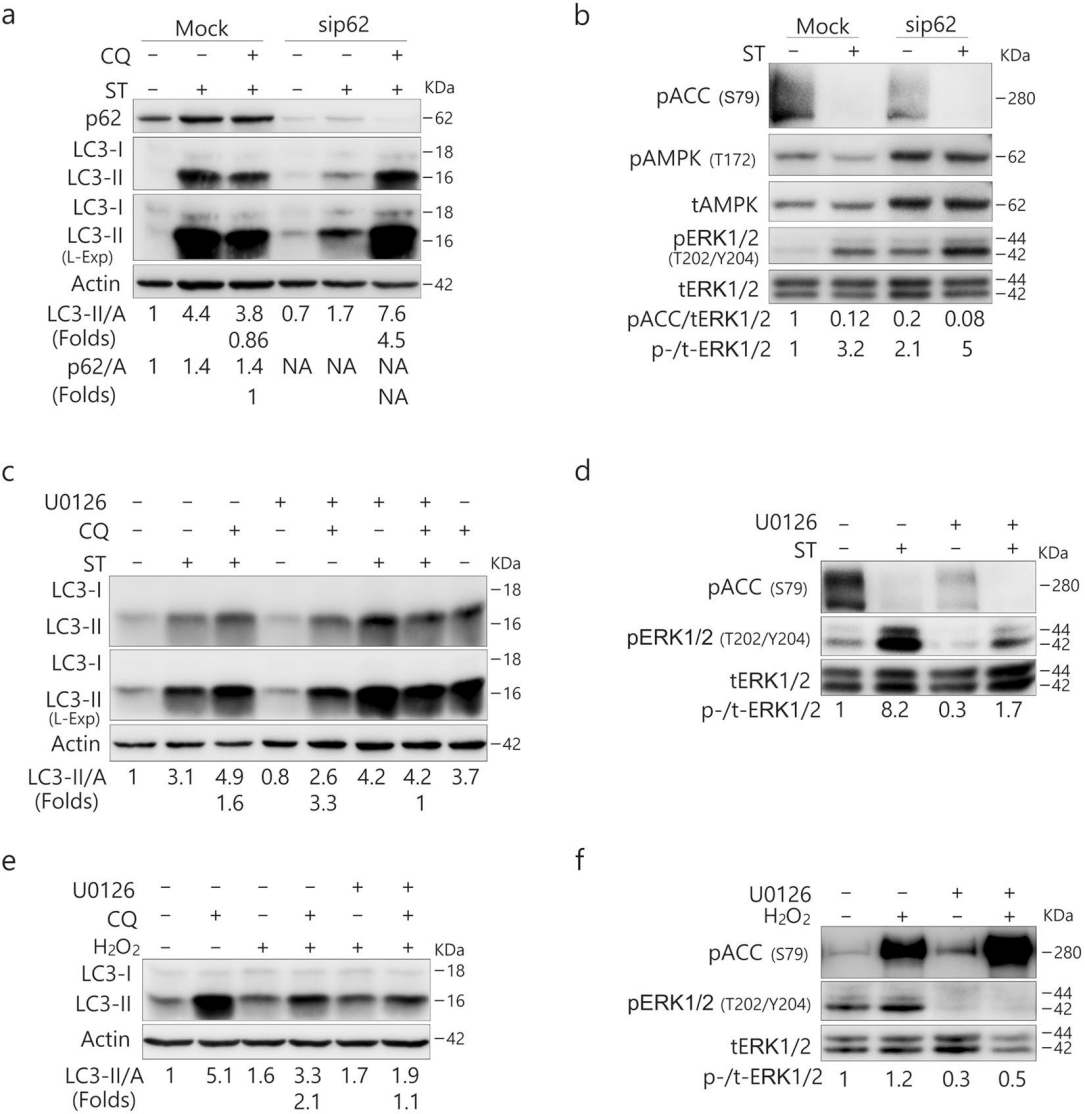
p62 deprivation reduces AMPK activity, and MEK/ERK inhibition suppresses the ST-induced autophagic flux. (**a**,**b**) 786-O cells were transfected with the p62 siRNAs (sc-29679) for 48 h. The lysates were analyzed by immunoblotting (8–13% PAGE) following ST treatment (8 μM/L) for 4 h in the presence or absence of CQ (20 μM/L). (**c**–**f**) 786-O cells were treated with ST (4 μM/L) or H_2_O_2_ (0.1 mM/L, 2 h) with or without U0126 (10 μM/L) up to 4 h in the presence or absence of CQ (20 μM/L). Cells were lysed and subjected to immunoblotting (8–13% PAGE) with the indicated antibodies. The results were similar among experiments repeated twice. (Actin: A)

Consistent with the aforementioned results (Fig. 4a), knockdown of p62 reversed the inhibitory effect of ST (8 μM/L) on autophagy in 786-O cells (Fig. 7a). A former study has shown that p62 loss leads to enhanced ERK activity^32^, and here we also found that p62 depletion increased both basal and ST-induced phosphorylation of ERK1/2 (Fig. 7b). Therefore, we speculated that p62 loss reversed the inhibitory effect of ST on autophagy by upregulating ERK1/2 signaling. To test this hypothesis, we employed U0126, a specific inhibitor of mitogen-activated protein kinase(MEK)/ERK signaling^33^, in the following experiments. While either ST or U0126 activated the autophagic process, their combination failed to induce autophagy because CQ was unable to accumulate LC3-II in ST/U0126-treated cells (Fig. 7c). As expected, U0126 inhibited both basal and ST-induced phosphorylation of ERK1/2 (Fig. 7d). Furthermore, U0126 suppressed the H_2_O_2_-induced ERK1/2 phosphorylation and attenuated the autophagic flux induced by H_2_O_2_ (Fig. 7e,f). Although U0126 attenuated the phosphorylation of ACC (Fig. 7d), it enhanced H_2_O_2_-induced ACC phosphorylation (Fig. 7f), suggesting the presence of a crosstalk between AMPK and ERK1/2.

### ST mediates the interaction between p62 and ERK1/2

**Figure 8:**
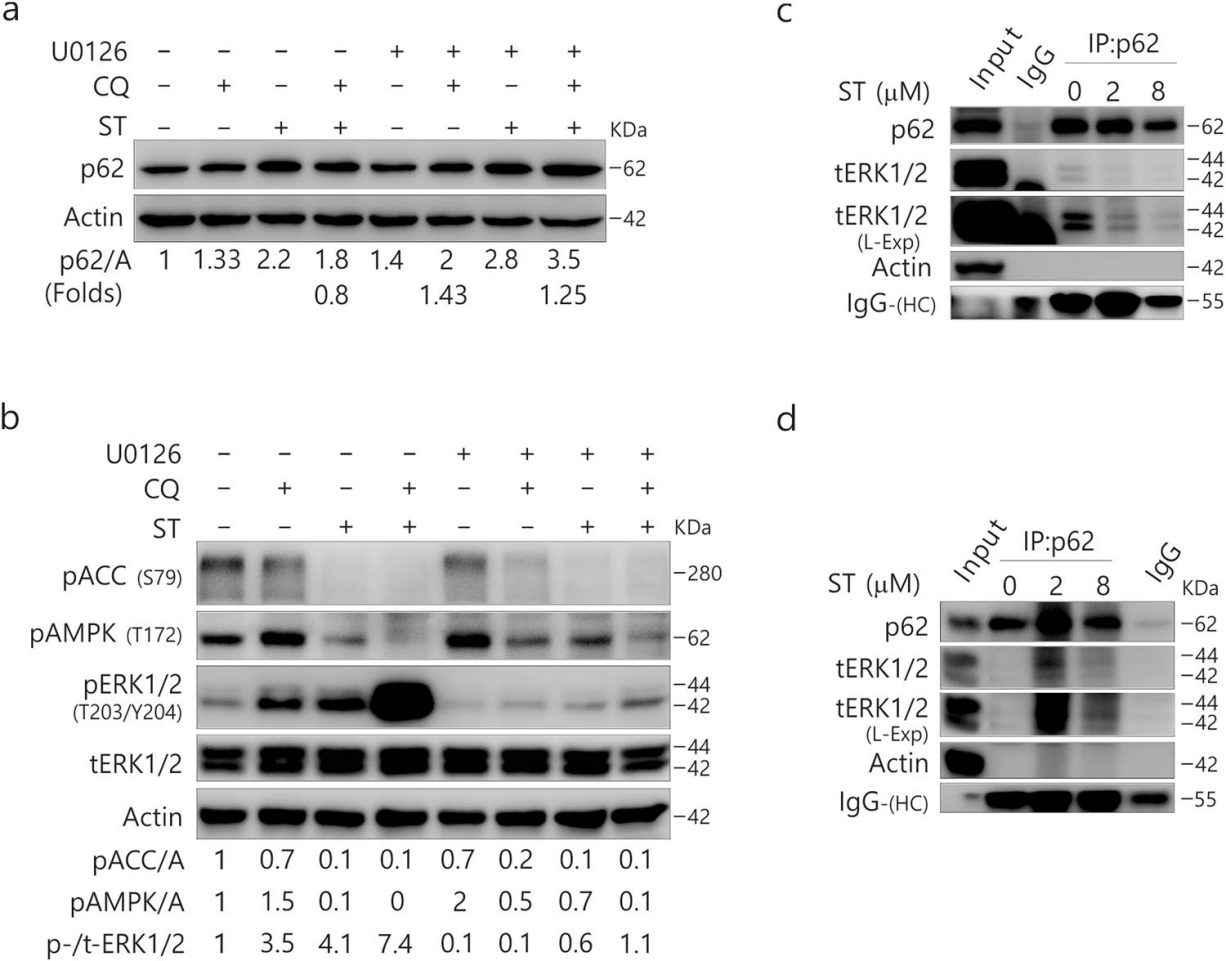
MEK/ERK could regulate the expression of p62. 786-O cells **(a**–**c)** and HeLa cells **(d)** were treated with ST (4 μM/L) with or without U0126 (10 μM/L) up to 4 h in the presence or absence of CQ (20 μM/L). Cells were lysed and subjected to immunoblotting (8–13% PAGE) and co-immunoprecipitated (8–13% PAGE) with the indicated antibodies. The results were similar among experiments repeated twice. (L-Exp: long exposure; HC: heavy chain; Actin: A)

Consistent with a former report^32^, we found that p62 could mediate the phosphorylation of ERK1/2, and thus we next examined whether MEK/ERK could regulate the expression of p62. Not only U0126 alone increased the levels of p62 but it further increased the levels of p62 in ST-treated cells (Fig. 8a). Although CQ alone increased the levels of p62 (Fig. 8a), it failed to further enhance the expression of p62 in ST- but not U0126-treated cells. CQ clearly failed to accumulate p62 in ST-challenged cells in relation to ERK1/2 signaling, as U0126 prevented the decrease in p62 in ST/CQ-treated cells (Fig. 8a,b). While CQ decreased the expression of p62 in ST-treated cells concurrent with a marked increase in ERK1/2 phosphorylation, U0126 inhibited the ST/CQ-induced phosphorylation of ERK1/2 and increased p62 levels in those cells (Fig. 8a,b). Consequently, these results may indicate a mutual regulatory relationship between p62 and phosphorylated ERK signaling, which may be related to the direct interaction between p62 and ERK1/2^34^.

To investigate whether p62 could interact with ERK1/2, we next carried out an immunoprecipitation assay using p62 antibody. Although we failed to pull-down phosphorylated-ERK1/2 in both HeLa and 786-O cells (data not shown), p62 interacted with total ERK1/2 (tERK1/2) in either 786-O or HeLa cells (Fig. 8c,d). Interestingly, while ST decreased the interaction between p62 and tERK1/2 in 786-O cells (Fig. 8c), its treatment increased the binding between these proteins in HeLa cells (Fig. 8d).

## Discussion

A new finding of the present study is that ST can either inhibit or induce autophagy even in the same cell line, depending on the expression of p62. Although ST was shown to suppress the induced phosphorylation of ACC, it failed to block H_2_O_2_-induced autophagic flux in p62-depleted cells. Therefore, p62 likely played a regulatory role in the inhibitory effect of activated AMPK on autophagy. Moreover, we revealed that ST regulated the interaction between p62 and ERK1/2 in a cell type-dependent manner.

It is well established that AMPK triggers autophagy through an indirect mechanism by inhibiting mTORC1 signaling or through direct binding to ULK1^20,21^. However, a recent study has shown that compound C, a commonly used inhibitor of AMPK, induces autophagy by blocking the Akt/mTOR pathway in a number of cancer cell lines. AMPK has also been reported to inhibit autophagy in isolated rat hepatocytes. Shang and Wang *et al*. revealed that nutrient starvation induces an acute autophagic process through ULK1 dephosphorylation and its dissociation from AMPK. They considered AMPK to have dual roles in the regulation of autophagy. Consistently, we found that ST (low dose) was able to induce autophagy concurrent with an inhibition of AMPK/ACC signaling, whereas H_2_O_2_ increased AMPK activity and triggered the autophagic process. Moreover, ST inhibited both H_2_O_2_-induced AMPK activity and autophagy. The aforementioned results confirmed that AMPK could have dual roles in regulating the autophagic process. Unexpectedly, we found that p62 loss reversed the inhibitory effect of ST on basal and H_2_O_2_-induced autophagy. Therefore, we reasoned that p62 was required for the activated AMPK to inhibit autophagy under certain conditions.

Despite its common use as a substrate in autophagy, growing evidence has indicated that p62 plays more active roles in the regulation of autophagy^35^. It has been demonstrated that p62 is required for RSV-induced autophagy, and RSV increases the expression of p62 mRNA and protein by mediating the activation of JNK (c-Jun N-terminal kinase)^10^. In addition, it has been reported that, to prevent oxidative liver damage, p62-dependent autophagy is required during the activation of Nrf2 (nuclear factor erythroid 2) by sestrins^36^. Consistently, we found that p62 was needed for H_2_O_2_ to stimulate the autophagic process, especially in 786-O cells. In p62-depleted HeLa cells, H_2_O_2_ at a dose of 0.5 mM/L failed to stimulate autophagy. Thus, p62 was able to regulate H_2_O_2_-induced autophagy relative to the stimulus dose. In contrast, p62 was unlikely to be required for ST-dependent autophagy, in which it actually appeared to play a negative regulatory role. Consequently, p62 could regulate autophagy in a stimulus- and cell-type dependent manner.

Consistent with former reports^37,38^, we found that p62 was able to mediate the phosphorylation of ERK1/2, which is a key member of the MAPK family and well established for its regulatory role in the autophagic process^39–41^. In the human colon cancer cell line HT29, ERK1/2 activation has been shown to promote macroautophagy and induce autophagic vacuolation^42^, and ERK activation is also needed for the starvation-induced autophagic process^43^. Lindane, a widely used environmental carcinogen, induces sustained phosphorylation of ERK and enhances the persistent formation of large autolysosomal vesicles, which can be inhibited by pretreatment with MEK1/2 inhibitors^44^. However, other studies have shown that oncogenic activation of Ras, the upstream activator of ERK, reduces autophagy in transformed cell lines^45,46^.

Our data showed that U0126 alone failed to inhibit the autophagic flux, whereas its treatment blocked or attenuated autophagy induced by either ST or H_2_O_2_. Thus, we considered MEK/ERK to potentially have differential regulatory roles in basal and induced autophagic processes. In fact, we observed that H_2_O_2_ treatment alone could induce autophagy concurrent with a downregulation of MEK/ERK signaling in HeLa cells, whereas ST blocked the H_2_O_2_-induced autophagic flux concomitantly with an increase in the phosphorylation of ERK1/2. Therefore, we speculated that a coordination might exist between AMPK and MAPK in the regulation of autophagy. This phenomenon is often found in various signaling pathways. For example, it is well known that Ras-ERK and PI3K-mTOR signaling can be either interplayed or compensated^47^. While AMPK enhances autophagy via directly phosphorylating ULK1^48^, however, a recent study has revealed that, through a negative feedback loop, the latter demonstrated to influence AMPK phosphorylation^49^. Additionally, one study showed that AMPK was able to either inhibit or enhance the PI3K signaling^50^. The data presented here clearly revealed that p62 could regulate the function of AMPK relating to the cell type, and it appeared to regulate ERK1/2 signaling in a time-dependent manner, implying that p62 could function either downstream or upstream of ERK signaling in a context-dependent manner. While ST together with CQ markedly increased the level of phosphorylated ERK1/2, their combination demonstrated to inhibit the expression of p62, whereas U0126 suppressed ERK1/2 phosphorylation and prevented p62 from loss in ST/CQ-treated cells. In contrast to former reports^34^, we failed to observe p62 pulled down with phosphorylated ERK; instead, p62 interacted almost equally with both ERK1 and ERK2 in 786-O cells. While ST increased the interaction between p62 and ERK1/2 in HeLa cells, it decreased the binding between these proteins in 786-O cells. Thus, the relationship between ERK1/2 and p62 may be much more complicated than previously understood.

## Conclusions

In summary, we show that p62 is responsible for the inhibition of ST in basal and H_2_O_2_-induced autophagy and reveal a previously unknown link between the activation of AMPK and the inhibition of autophagy. Future work in this direction will enable us to better understand the regulatory mechanism of autophagy, illuminate the molecular mechanism of ST resistance in cancer treatment and provide a resolution to dealing with the devastating effects of anti-angiogenesis resistance.

## Methods

### Chemicals and antibodies

Sunitinib (ST; S126061) was purchased from Aladdin (Seattle, WA, USA). 3-methyladenine (3-MA; M9281), chloroquine diphosphate salt (CQ; C6628), U0126 monoethanolate (u120), AICAR (A9978) and polyclonal antibodies against LC3 (L7543) were acquired from Sigma-Aldrich (St. Louis, MO, USA). H_2_O_2_ (E882) was purchased from Amresco (WA, USA). The antibodies against PARP- (9542), Phospho-AMPKα (Thr172; 2535), AMPKα (2532), Phospho-p44/42MAPK (ERK1/2; Thr202/Tyr204; 9106), Phospho-Acetyl-CoA Carboxylase (Ser79; 3661), p44/42 MAPK (total-ERK1/2; 9102) were obtained from Cell Signaling Technology (Boston, MA, USA). The antibody against LC3 (M152-3) for immunoprecipitation was purchased from Medical & Biological Laboratories (Naka-ku, Nagoya, Japan). The antibody against p62 (18420-1-AP) was bought from Proteintech (Wuhan, Hubei, China). The antibody against actin (TA-09) was acquired from ZhongShanJinQiao Biocompany (Beijing, China). MTS reagent powder (G1111) was obtained from Promega Corporation (Madision, WI, USA). Alexa Fluor 594 goat anti-rabbit IgG (H + L) (R37117) and Alexa Fluor 488 goat anti-mouse IgG (H + L) (A-11001) were purchased from Molecular Probes (Eugene, OR, USA).

### siRNAs

The siRNA specific for human MAP LC3α (sc-106197), SQSTM1/p62 (sc-29679), and BECN1 (sc-29797) were purchased from Santa Cruz Biotechnology (Dallas, TX, USA), along with the control siRNA (sc-37007). The siRNA specific for human MAP1LC3B (81631), SQSTM1/p62 (8878), and BECN1 (8678) were purchased from Dharmacon Biotechnology (Lafayette, CO, USA), along with the control siRNA (D-001136-01).

### Cell culture and immunoblotting analysis

786-O, ACHN, and HeLa cells were grown in DMEM media containing 10% fetal bovine serum (GIBCO, Grand Island, NY, USA) with 1% antibiotics. Cells were cultured overnight to grow about 70–80% confluency before treated with compounds. For siRNA interference, about 30% confluence cells in the media without antibiotics were transfected using DharmaFECT (Dharmacon, T2001) according to the manufacturer’s instructions. After transfection for 48 h, Cells were split and cultured overnight before treatments. Whole cell lysate was prepared with lysis using Triton X-100/glycerol buffer, containing 50 mM Tris-HCl (pH 7.4), 4 mM EDTA, 2 mM EGTA, and 1 mM dithiothreitol, supplemented with 1% Triton X-100, 1% SDS, and protease inhibitors, and then separated on a SDS-PAGE gel and transferred to PVDF membrane. Immunoblotting was performed using appropriate primary antibodies and horseradish peroxidase-conjugated suitable secondary antibodies, followed by detection with enhanced chemiluminescence (Pierce Chemical Rockford, IL, USA)^51–53^.

### Immunoprecipitation

Whole cell lysate was prepared with lysis using Triton X-100/glycerol buffer as mentioned above. LC3 or p62 was immunoprecipitated using the corresponding antibody at 4 °C for 3 h, and then incubated with Protein G-Sepharose (Vigorous Biotechnology Beijing, China) for 1 h. Immunoprecipitates and cell lysates were electrophoresed on SDS-PAGE and subjected to immunoblotting analysis^53^.

### Cell viability assay (MTS)

Cells were cultured in 96-well plates (7,500 cells per well) with 100 µL complete culture media. After overnight culturing, cells were replaced with Phenol red free complete medium which was added with either drug-free or ST or other chemicals. Cells were cultured for indicated period and the cell viability was detected by CellTiter 96 Aqueous Non-Radioactive Cell Proliferation Assay (Promega)^51,52^.

### Electron microscopy

Wash the samples three times with PBS, trypsinize, and then centrifuge to collect them. Fix the cell pellets with 4% paraformaldehyde at 4 °C overnight, post-fix them with 1% OsO4 in cacodylate buffer at RT for 1 h, and then dehydrate stepwise with ethanol. Rinse the dehydrated pellets with propylene oxide at RT for 30 min and embed them in Spurr resin for sectioning. Finally, use a transmission electron microscope (JEM1230 Akishima, Tokyo, Japan) to observe the images of the thin sections^51,53^.

### RNA extraction and qPCR analysis

The total cellular RNA was extracted using TRIzol reagent (Invitrogen, Carlsbad, CA, USA; 15596-018) according to the manufacturer’s protocol, and 1 μg of RNA was reversely transcribed at 42 °C for 60 min in 20 μL PrimeScriptTM RT reagent Kit (TaKaRa, Dalian, Liaoning, China; DRR037A). Reactions were stopped by heat inactivation at 85 °C for 5 s^51–53^. Primer sequences used for amplification were as follows (Table 1):

qPCR (CFX96^TM^; Bio-Rad) was initiated with a 10 min denaturation at 95 °C in a final volume of 20 μL. The cycle profile was 95 °C (15 s), 60 °C (45 s) and 72 °C (1 min.) for up to 40 cycles. The data were calculated based on the internal control of β-actin.

### Fluorescence microscopy

Cells were plated on glass cover slips and the indicated treatments were performed. Cells were washed with Ca^2+^- and Mg^2+^-free PBS (CMF-PBS), fixed with freshly prepared 4% paraformaldehyde at 4 °C for 30 min and permeabilized incubation with CMF-PBS containing 0.1% TritoX-100 and 0.5% BSA at RT for 5 min. Cells were then washed three times with CMF-PBS, blocked in CMF-PBS containing 3% BSA for 1 h, and incubated with the indicated antibodies in the presence of 0.1% TritoX-100 and 0.5% BSA. After washing three times, cells were then stained with Alex Fluor 488 or 594 secondary antibodies for 1 h. Cells were then immersed in VECTASHIELD with DAPI (H1200) to visualize the nuclei after washing three times. Images were acquired via Fluorescence microscopy (Zeiss Heidenheim, Germany)^53^.

### Statistical analysis

The images were analyzed to verify the linear range of chemiluminescence signals and quantifications were carried out using densitometry. The normally distributed data are shown as mean ± SD and analyzed using one-way analysis of variance and the Student-Newman-Keuls post-hoc test. Data are shown as Mean ± SD in Graphs. A P-value < 0.05 was considered to have significant differences.

## Supplementary information

Supplemental Information

## Data Availability

All data and materials are available.
