# Supplementary material for: SQSTM1/p62 loss reverses the inhibitory effect of sunitinib on autophagy independent of AMPK signaling
Source: Sci Rep. 2019 Jul 31;9:11087. doi: 10.1038/s41598-019-47597-4 (PMC6668422; doi:10.1038/s41598-019-47597-4)
Supplement: Supplementary file 1 — Supplemental Information [file 41598_2019_47597_MOESM1_ESM.pdf]

SQSTM1/p62 loss reverses the inhibitory effect of sunitinib on autophagy independent of AMPK signaling

Bolin Hou,[1,2†] Gang Wang,[3†] Quan Gao,[1,2†] Yanjie Wei,[1,2] Caining Zhang,[1,2] Yange Wang,[1,2] Yuqing Huo,[4] Huaiyi Yang,[5] Xuejun Jiang[1,*] and Zhijun Xi[3,*]

[1] State Key Laboratory of Mycology, Institute of Microbiology, Chinese Academy of Sciences, Beijing 100101, China

[2] University of Chinese Academy of Sciences, Beijing 100039, China

[3] Department of Urology, Peking University First Hospital, Beijing 100034, China

[4] Vascular Biology Center, Department of Cellular Biology and Anatomy, Medical College of Georgia, Augusta University, Augusta 30912, Georgia, USA

[5]CAS Key Laboratory of Pathogenic Microbiology and Immunology, Institute of Microbiology, Chinese Academy of Sciences, Beijing 100101, China

[†]These authors contributed equally to this work

*Correspondence to: Zhijun Xi and Xuejun Jiang; Email: xizhijun@hsc.pku.edu.cn and jiangxj@im.ac.cn

Contents: **Supplementary Figures (S1, S2 and S3)**

Adequate doses of CQ selection

Scanned raw images for the western blot

Supplementary Figure S1

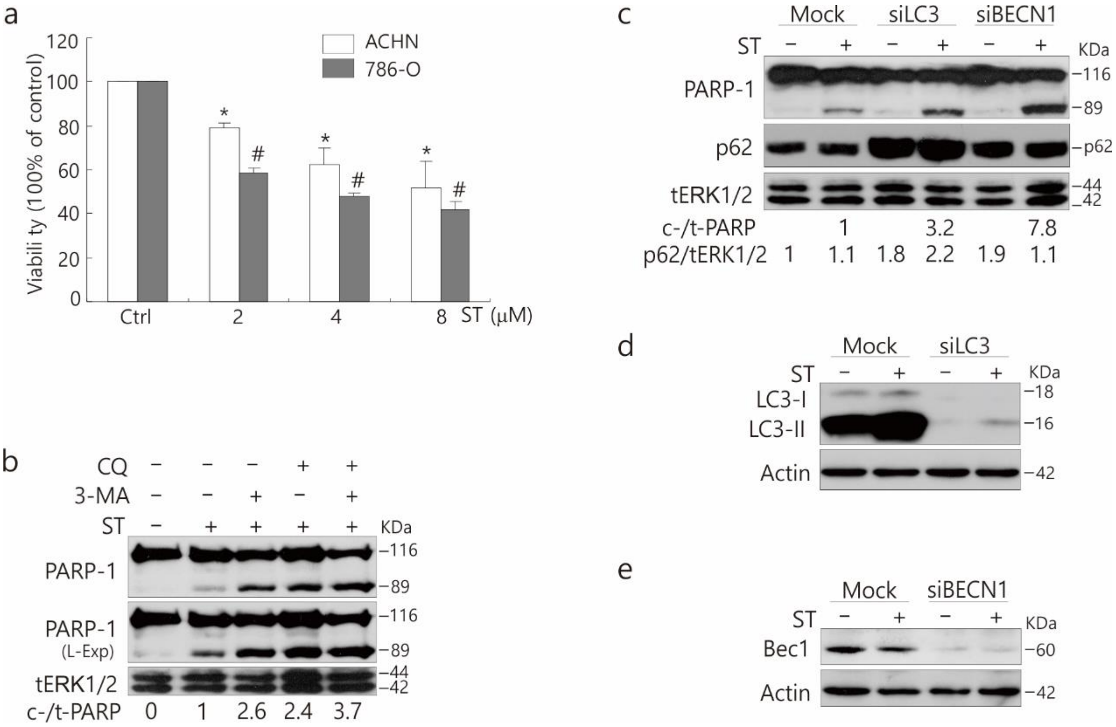

Supplementary Figure S1. Inhibition of autophagy increases ST-induced cleavage of PARP-1. (**a**) 786-O and ACHN cells were treated with ST (0–8 μM/L) for 48 h; cell viability was analyzed by the MTS assay as described in Materials and Methods. Data are presented as the mean ± S.D. and are representative of three independent experiments. Ctrl: cells with an equal amount of DMSO. (**b**) 786-O cells were treated with ST (8 μM/L) or together with 3-MA (1 mM/L), or CQ (15 μM/L) for 8 h, and the cells were lysed and subjected to immunoblotting with the indicated antibodies; tERK1/2 was used as a loading control. Densitometry was performed for quantification, and the relative ratios of cleaved PARP-1 (cPARP-1) are shown below the blots. (**c-e**) 786-O cells were transfected with the indicated siRNAs (which were purchased from Santa Cruz Biotechnology) for 48 h. The lysates were analyzed by

immunoblotting with the indicated antibodies following ST (8 μM/L) for 48 h. The results were similar among experiments repeated at least three times. (L-Exp: long exposure; Actin: A)

Supplementary Figure S2

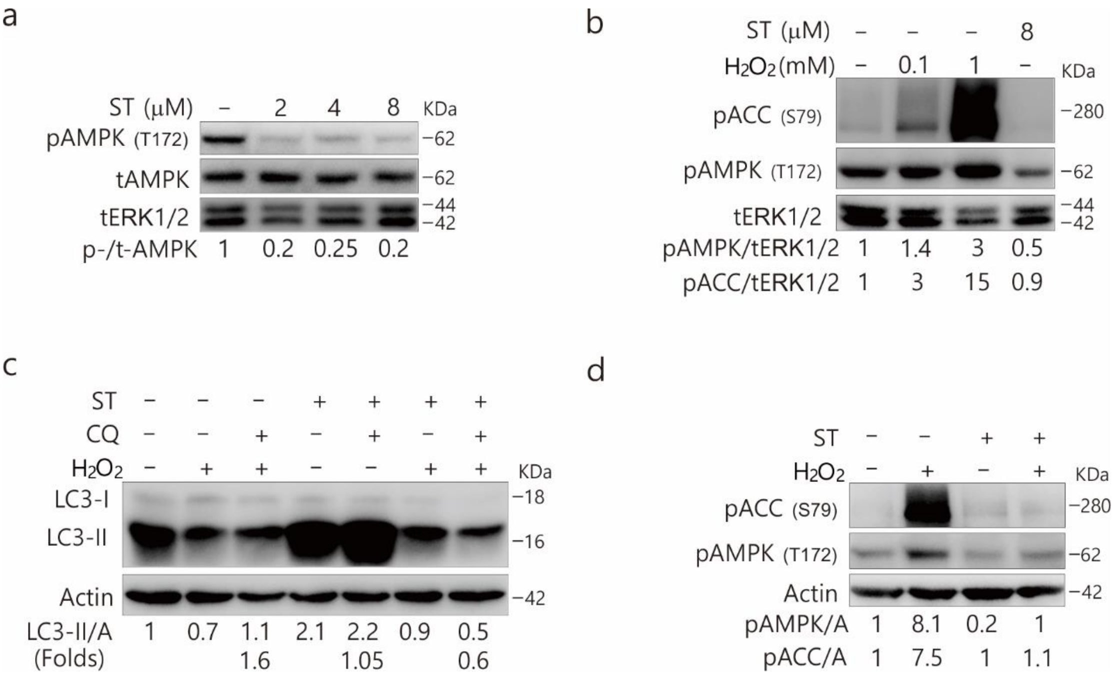

Supplementary Figure S2. ST inhibits the phosphorylation of AMPK and ACC and blocks H_2O_2-induced autophagy concurrent with the downregulation of AMPK activity. HeLa (**a** and **b**) cells were treated with ST (0–8 μM/L) or H_2O_2 (0.1, 1 mM/L; 2 h) for up to 4 h, and the cells were lysed and subjected to immunoblotting with the indicated antibodies. tERK1/2 was used as a loading control. The results were similar among experiments repeated at least three times. HeLa (**c** and **d**) cells were treated with H_2O_2 (0.5 mM/L) with or without ST (8 μM/L), or AICAR (0.5 mM/L) in the presence or absence of CQ (15 μM/L) for 2 h. Cells were lysed and subjected to immunoblotting with the indicated antibodies. Actin was used as the loading control.

The results were similar among experiments repeated at least twice. (Actin: A)

Supplementary Figure S3

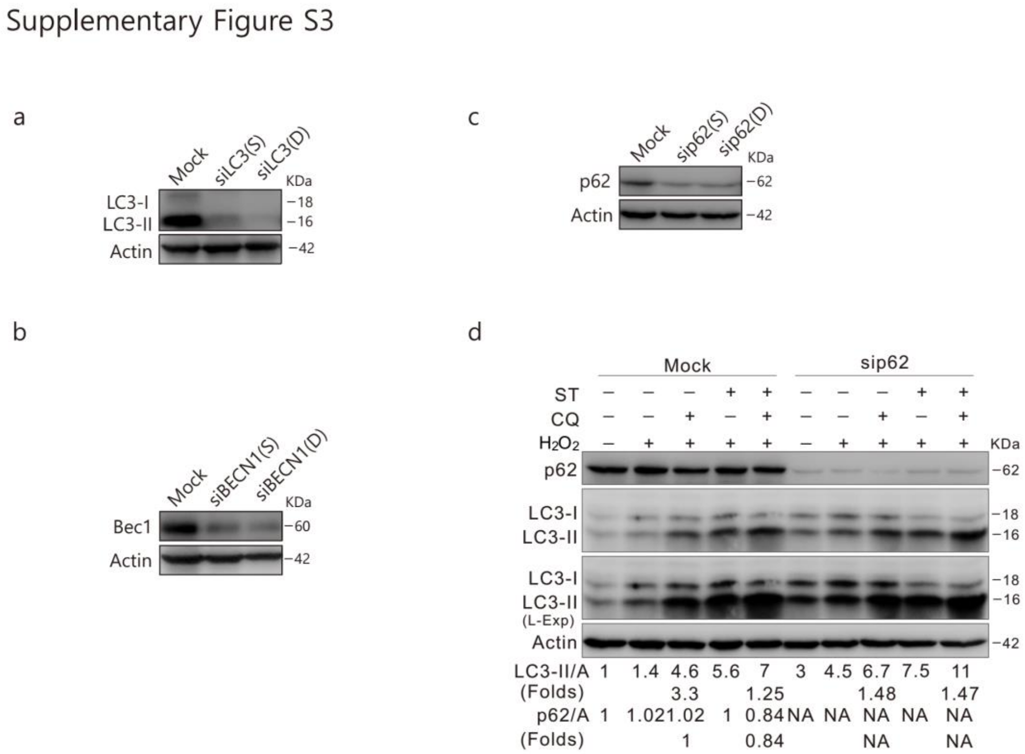

Supplementary Figure S3. Depletion of p62 inhibits H_2O_2-induced autophagy and reverses the inhibitory effect of ST on H_2O_2-dependent autophagic flux. (**a-c**) 786-O cells were transfected with the indicated siRNAs (purchased from Santa Cruz Biotechnology (S) and Dharmacon Biotechnology (D), respectively) for 48 h. The lysates were analyzed by immunoblotting with the indicated antibodies. 786-O cells (**d**) were transfected with p62 siRNAs (8878) for 48 h. The lysates were analyzed by immunoblotting following treatment with H_2O_2 (a, b: 0.1 mM/L) with or without ST (8 μM/L) for 2 h in the presence or absence of CQ (20 μM/L). The results were similar among experiments repeated twice. (Actin: A; Beclin1: Bec1; L-Exp: long exposure)

Adequate doses of CQ

Generally, there two methods to detect autophagic flux (autophagy), one is using the ratios between LC3-II and LC3-I; the other one is to examine autophagy in the presence of autophagosome-lysosome fusion inhibitor, such as CQ or Bafilomycin A1 (Baf A1).Our laboratory has done experiments in the field of autophagy since 2006, the concentration of CQ has been used in this manuscript is based on the optimizing in different cell lines as the results of immunoblotting show below.

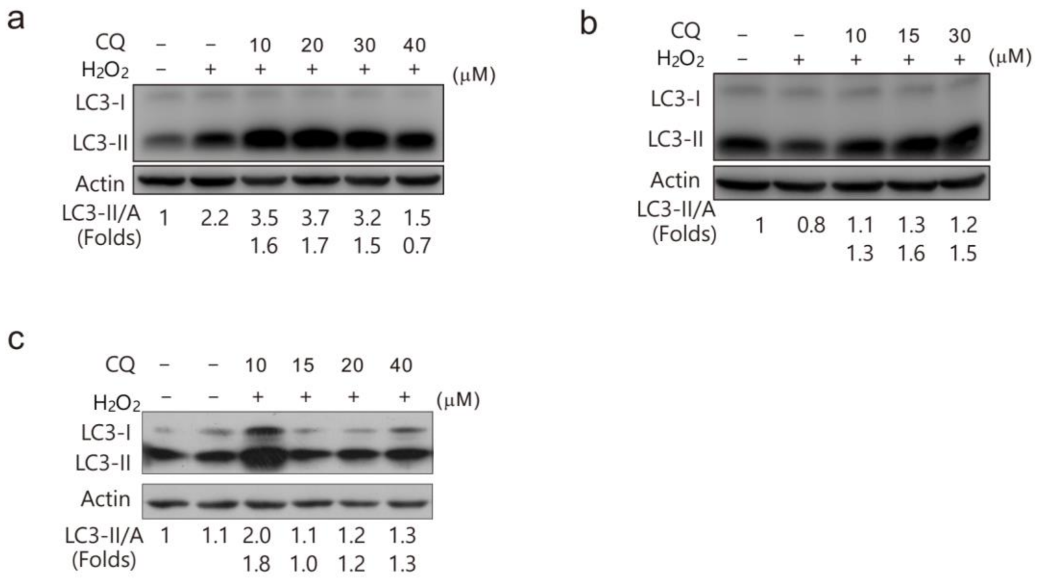

a: 786-O cells.

b: HelaL cells.

c: ACHN cells.

These results show that the adequate concentration of CQ is between 10 and 20 μM.

The following are some our publications in which CQ has been used to detect autophagy flux.

1. Niu S, Yuan D, Jiang X, Che Y.11'-Deoxyverticillin A (C42) promotes autophagy

through K-Ras/GSK3 signaling pathway in HCT116 cells. *Protein Cell* 2014, 5(12):945-949

2. Lu Q, Yan S, Sun H, Wang W, Li Y, Yang X, et al. Akt inhibition attenuates rasfonin-induced autophagy and apoptosis through the glycolytic pathway in renal cancer cells. *Cell death & disease* 2015, 6: e2005.
3. Yan S, Liu L, Ren F, Gao Q, Xu S, Hou B, *et al.* Sunitinib induces genomic instability of renal carcinoma cells through affecting the interaction of LC3-II and PARP-1. *Cell death & disease* 2017, **8**(8)**:** e2988.
4. Yan SY, Wei XL, Xu SS, Sun H, Wang WJ, Liu L, *et al.* 6-Phosphofructo-2-kinase/fructose-2,6-bisphosphatase isoform 3 spatially mediates autophagy through the AMPK signaling pathway. *Oncotarget* 2017, **8**(46)**:** 80909-80922.
5. Gao Q, Hou B, Yang H, Jiang X. Distinct role of 4E-BP1 and S6K1 in regulating autophagy and hepatitis B virus (HBV) replication. *Life sciences* 2019, **220:** 1-7.

Scanned raw images for the western blot

Raw Figure1

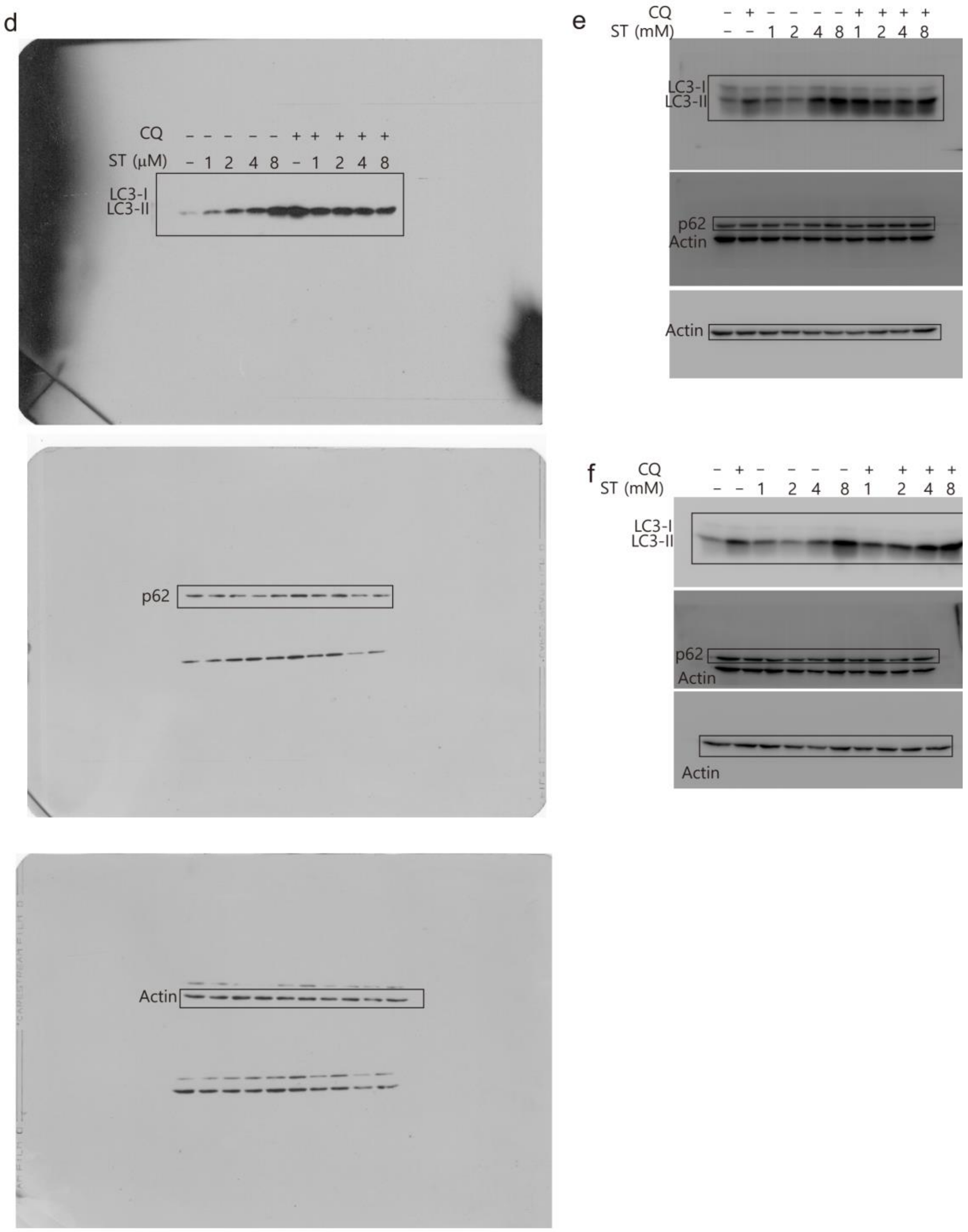

PVDF membranes were cut into stripes for different antibodies staining. d and e or f were exposed by X-ray and Thermo fisher Luminometer.

Raw Figure2

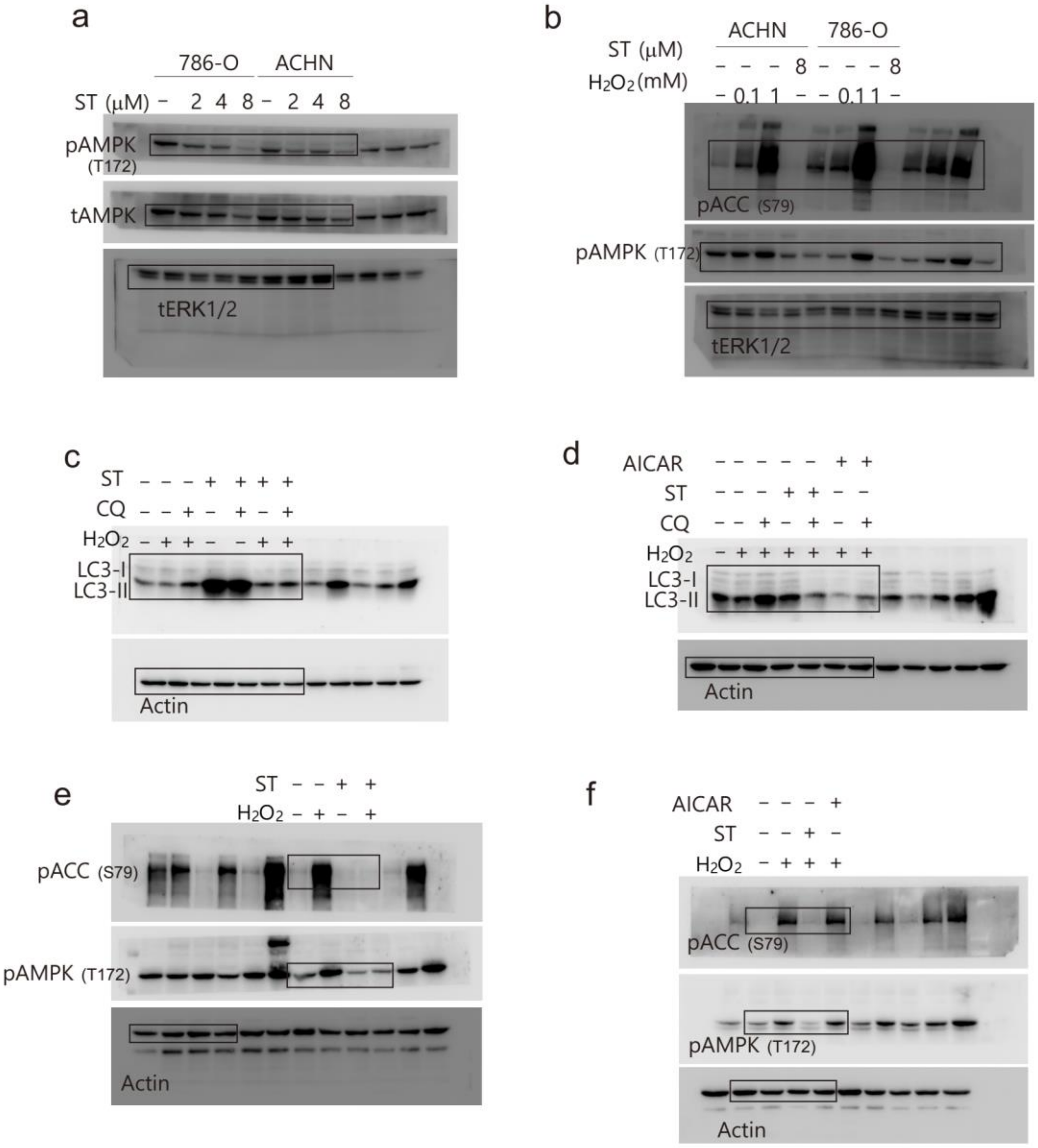

PVDF membranes were cut into stripes for different antibodies staining. All images were exposed by Thermo fisher Luminometer. Figure2 e is the result of the same batch of samples running with SDS-PAGE on different days.

Raw Figure3

a and b

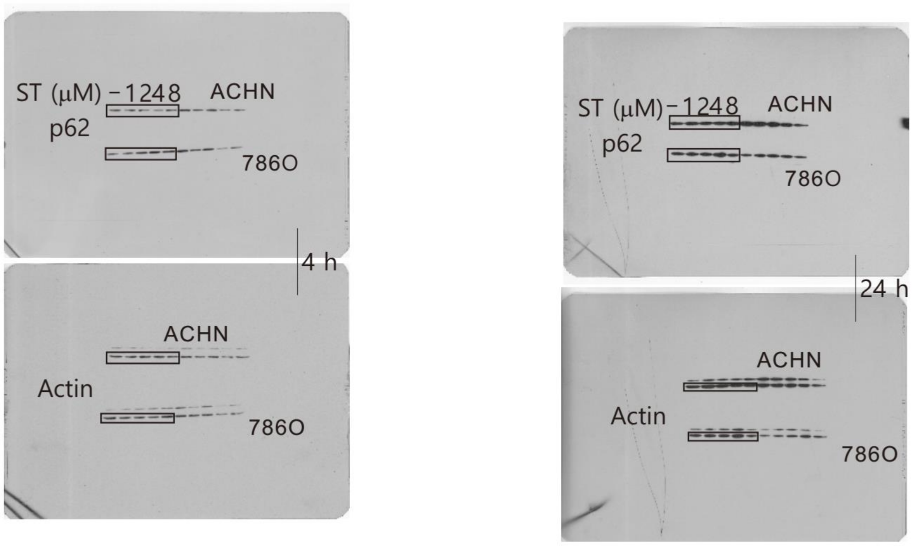

e

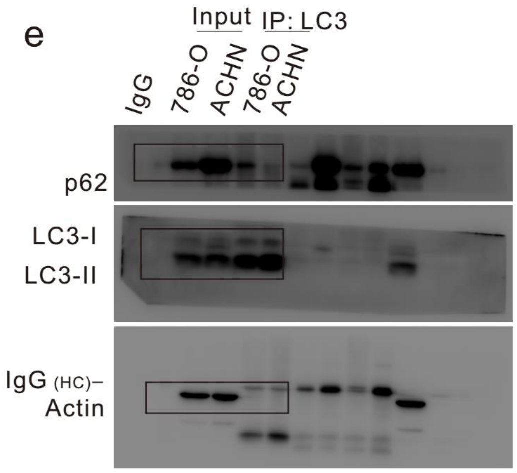

f

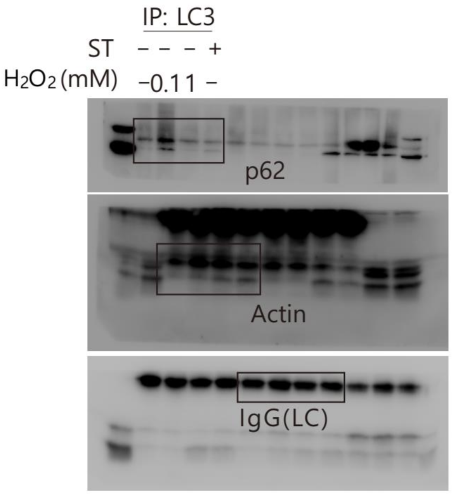

PVDF membranes were cut into stripes for different antibodies staining. a and b were exposed by X-ray, e and f were exposed by Thermo fisher Luminometer. Figure3 f is the result of the same batch of samples running with SDS-PAGE on different days.

Raw Figure4

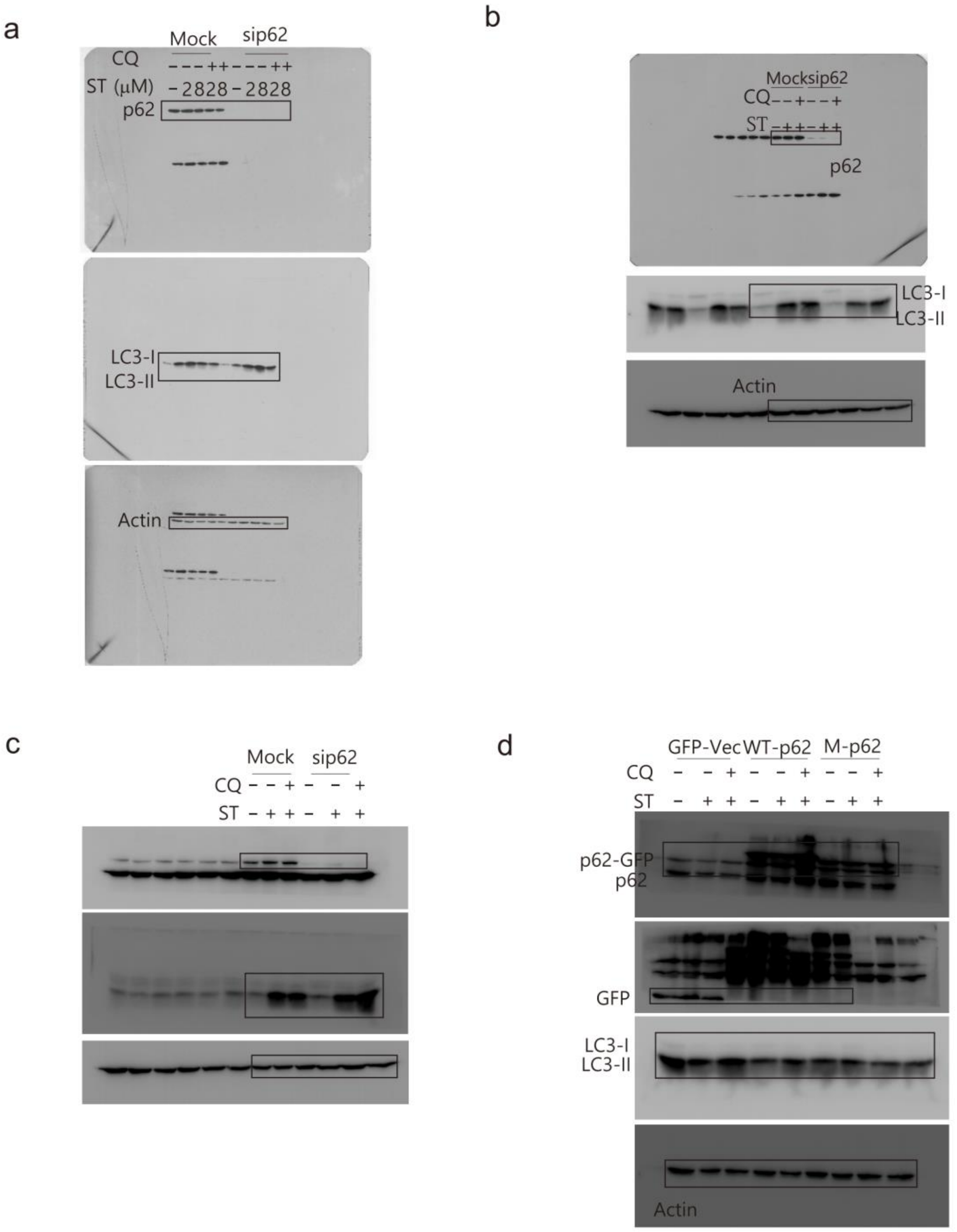

PVDF membranes were cut into stripes for different antibodies staining. a and b were exposed by X-ray, c and d were exposed by Thermo fisher Luminometer. Figure4 d is the result of the same batch of samples running with SDS-PAGE on different days

Raw Figure5

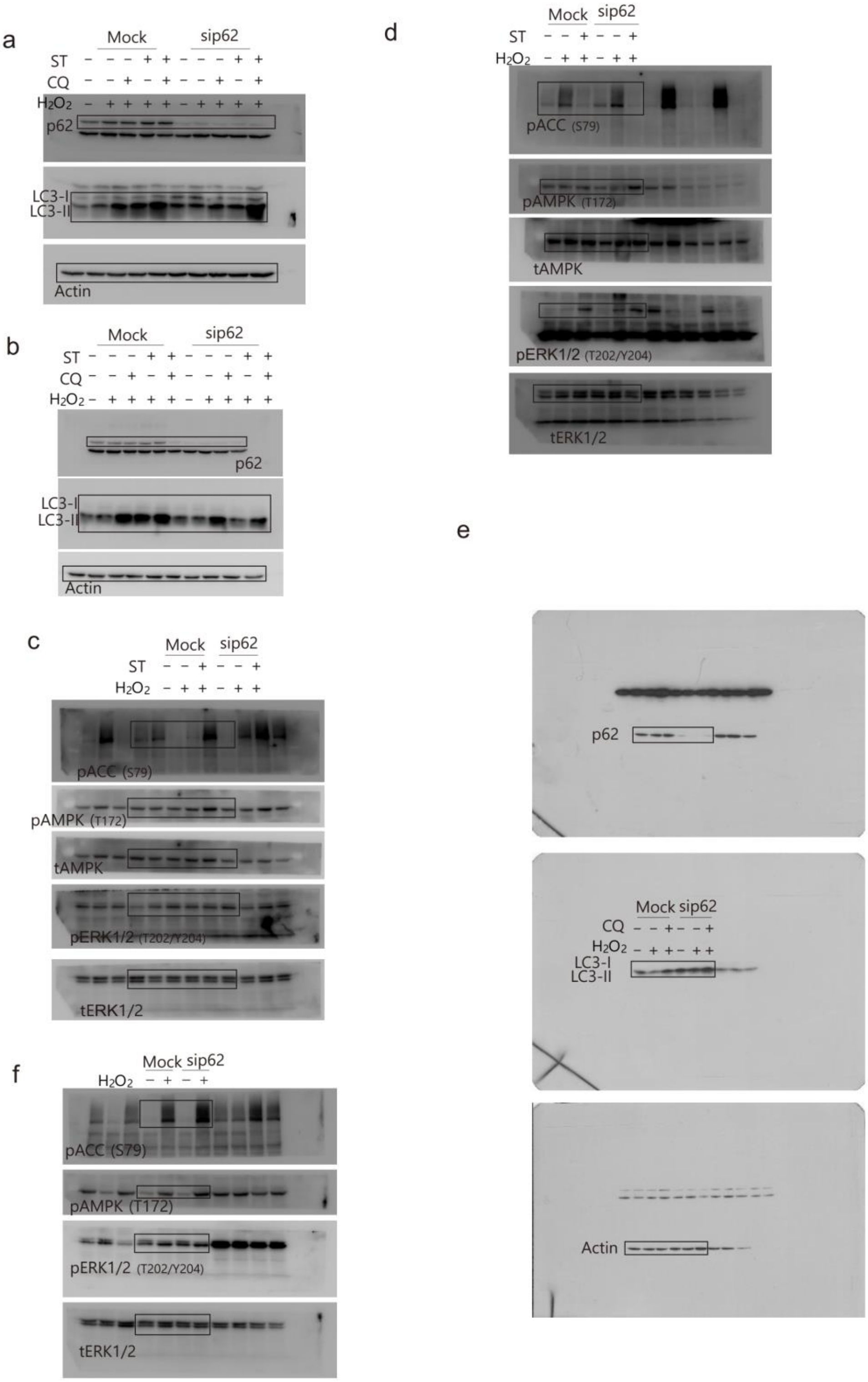

PVDF membranes were cut into stripes for different antibodies staining. Figure5 e was exposed by X-ray, others were exposed by Thermo fisher Luminometer.

Raw Figure6

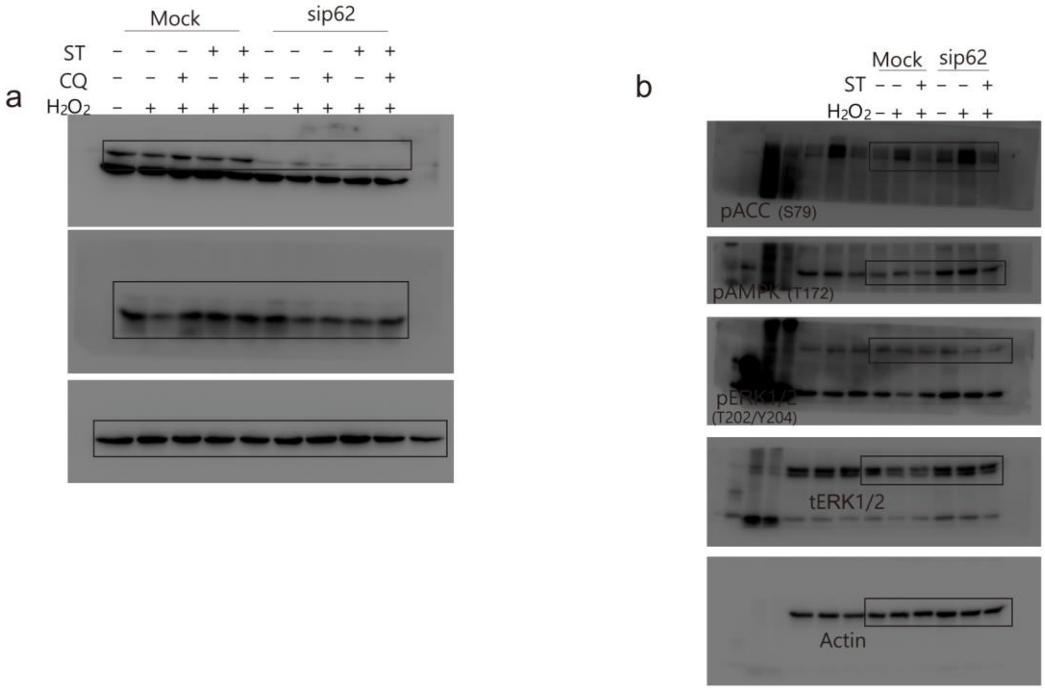

PVDF membranes were cut into stripes for different antibodies staining. All images were exposed by Thermo fisher Luminometer.

Raw Figure7

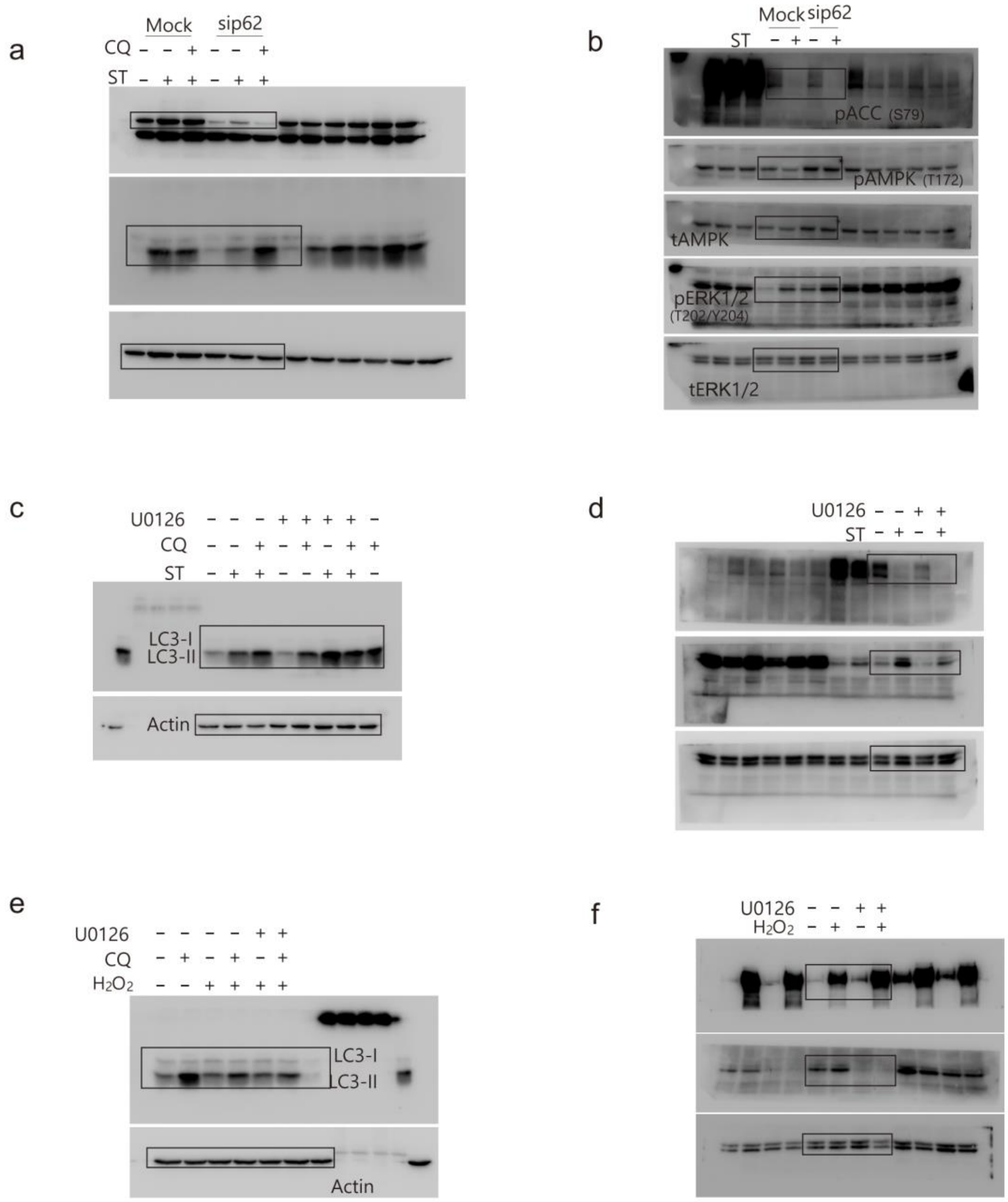

PVDF membranes were cut into stripes for different antibodies staining. All images were exposed by Thermo fisher Luminometer.

Raw Figure8

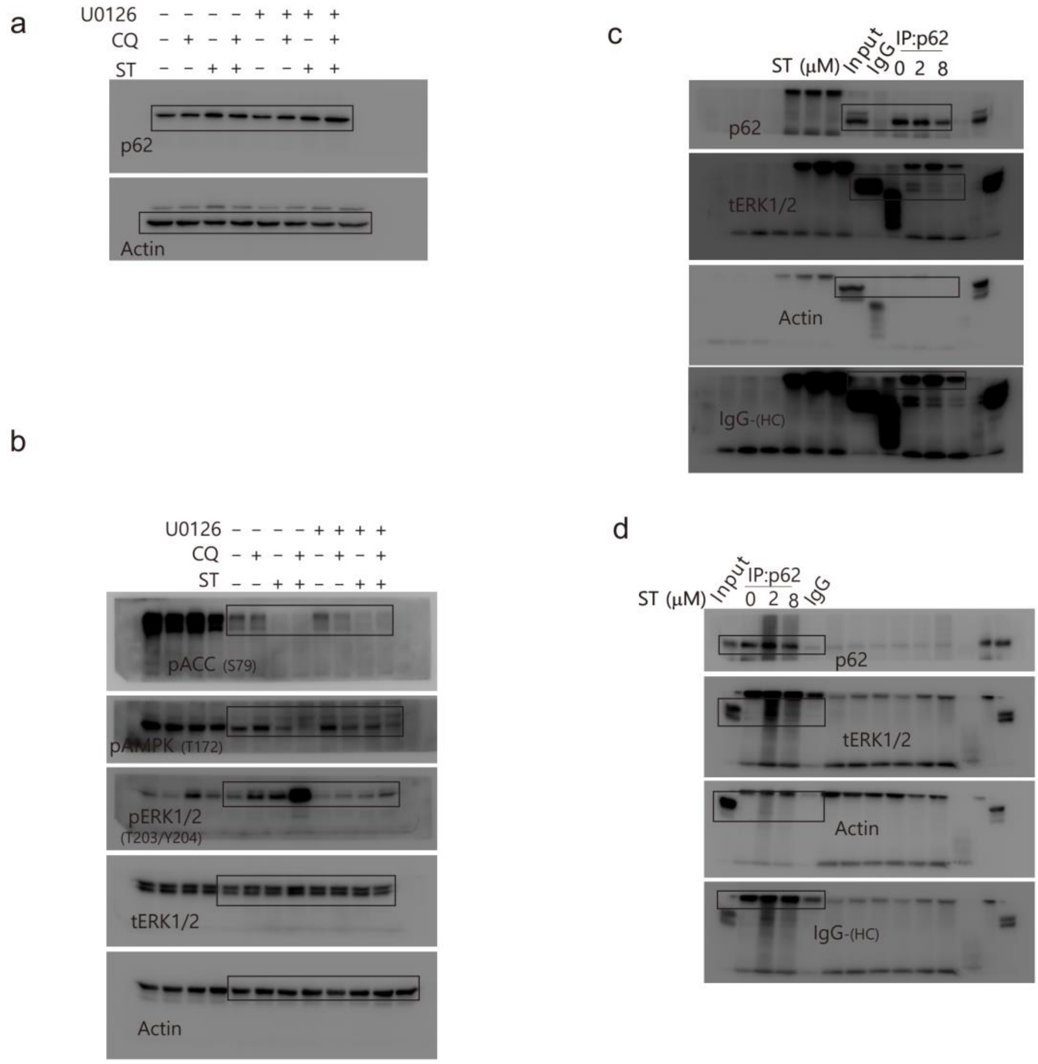

PVDF membranes were cut into stripes for different antibodies staining. All images were exposed by Thermo fisher Luminometer.

Raw Supplemental Figure1

b

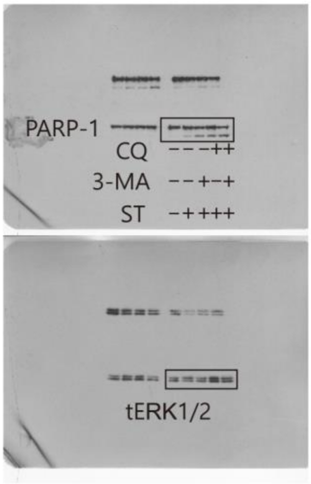

c

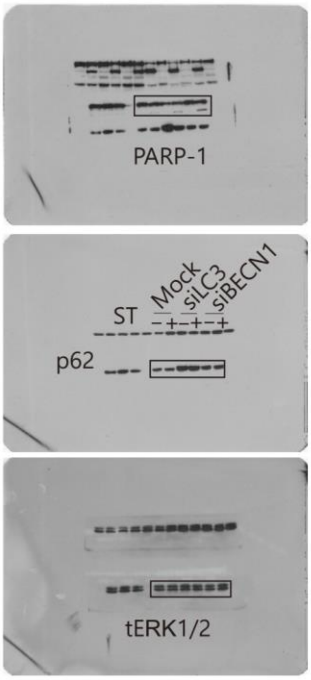

d

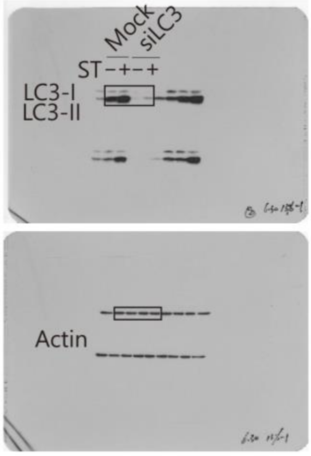

e

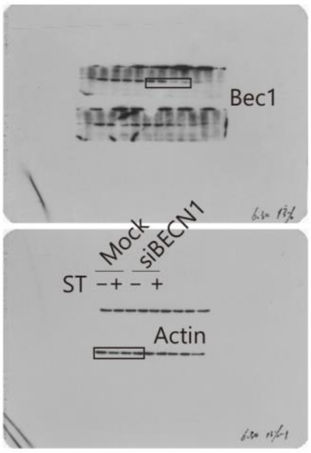

PVDF membranes were cut into stripes for different antibodies staining. All images were exposed by X-ray. Supplemental Figure1 e is the result of the same batch of samples running with SDS-PAGE on different days.

Raw Supplemental Figure2

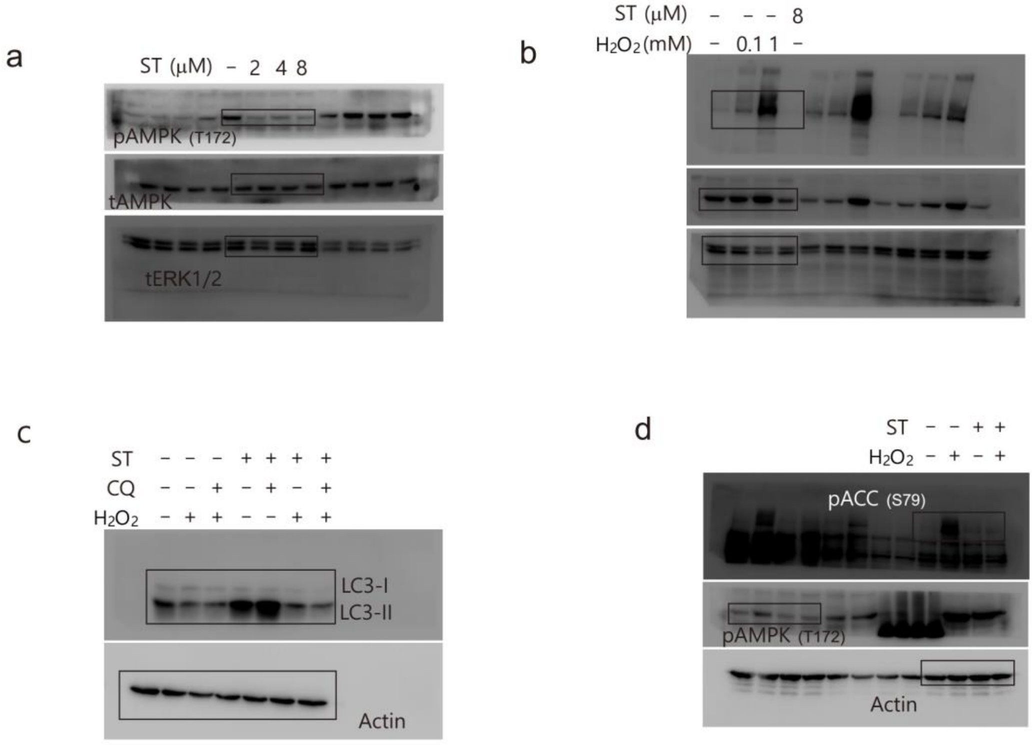

PVDF membranes were cut into stripes for different antibodies staining. All images were exposed by Thermo fisher Luminometer. Supplemental Figure2 d is the result of the same batch of samples running with SDS-PAGE on different days.

Raw Supplemental Figure3

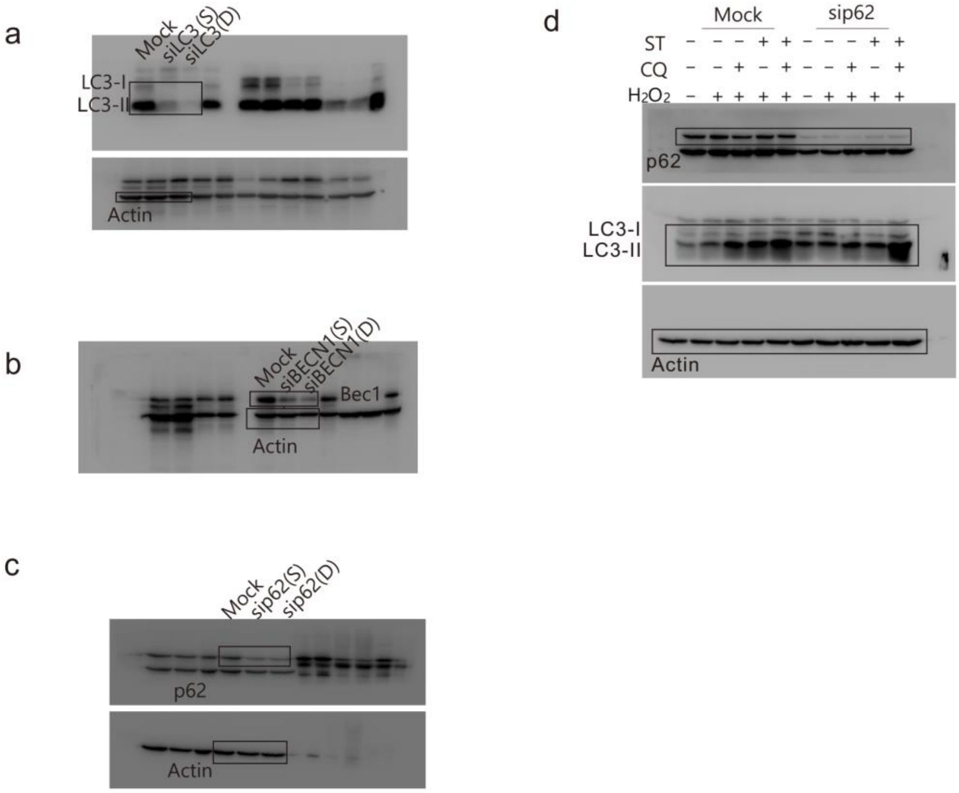

PVDF membranes were cut into stripes for different antibodies staining. All images were exposed by Thermo fisher Luminometer.
